# Bioactive Constituents and Therapeutic Mechanisms of Shenfu Decoction in a Rat Model of Seawater-Immersion-Induced Accidental Hypothermia

**DOI:** 10.3390/ph19050793

**Published:** 2026-05-19

**Authors:** Yanrong Gong, Zhibo Wang, Yiwen Ben, Hongzhi Chen, Yajing Wang, Chaoyue Sun, Huifang Deng, Huiqing Zhang, Zifei Yin, Wei Gu

**Affiliations:** 1 School of Traditional Chinese Medicine, Naval Medical University, Shanghai 200433, China; 2 Basic Medicine College, Naval Medical University, Shanghai 200433, China

**Keywords:** seawater immersion, hypothermia, network pharmacology, survival analysis, uncoupling protein 1

## Abstract

**Background/Objectives**: Shenfu Decoction (SFD) is a traditional Chinese herbal formula composed of *Panax ginseng* and *Aconitum carmichaelii* that can revive and counteract shock. However, how SFD can mitigate hypothermia caused by seawater immersion is poorly understood. **Methods**: Three commonly used ratios of SFD (*Panax ginseng*:*Aconitum carmichaelii* = 1:1, 1:2, 2:1) were prepared, and their chemical properties were analyzed with UPLC-Q-TOF-MS. A rat model of hypothermia caused by seawater immersion at 15 °C was utilized. Survival analysis was used to evaluate the prophylactic effect of single intragastric administration of SFD with different ratios and doses on the survival time of rats, and to identify the optimal intervention conditions. Network pharmacology analysis based on the absorbed constituents of SFD was performed to preliminarily predict the underlying mechanisms, which were subsequently validated using RT-PCR, Western blotting, ELISA, and H&E staining. **Results**: SFD contained 54 compounds, including ginsenosides and aconitine alkaloids, whose relative concentrations varied across different ratios of SFD. Animal studies showed that pretreatment of SFD (1:1) administered at a dose of 1.35 g/kg was very effective in increasing rats’ survival time in hypothermia and slowed down core body temperature decline. Based on the 28 plasma-absorbed compounds of SFD, network pharmacology identified 503 targets, enriched in cAMP and MAPK signaling pathways. SFD (1:1, 1.35 g/kg) resulted in larger lipid droplets in brown adipose tissue (BAT) and enhanced the respiratory metabolic rate in seawater-immersion-induced hypothermia rats. Furthermore, its thermogenic effect is likely associated with the upregulation of uncoupling protein 1 (UCP1) via activating p38 MAPK/PGC1α/PPARγ and NE-(β3-AR)-cAMP-PKA pathways. **Conclusions**: The results of this study demonstrate that a single prophylactic administration of the traditional Chinese medicine formula SFD prior to cold seawater exposure significantly prolongs the survival time of rats. This effect is associated with the upregulation of UCP1 and the subsequent enhancement of thermogenesis in BAT. These findings highlight the great potential of SFD as a promising intervention for the management of hypothermia.

## 1. Introduction

Accidental hypothermia is a drop in core body temperature (CBT) below 35 °C and has a high risk of mortality [1,2,3]. Temperature relation analysis on 272 major cities in China indicates that low temperatures can cause non-accidental death [4]. Another study shows that from 1960 until 2020, cold waves in China caused approximately 1.133 million deaths [5]. In addition, an international collaborative study showed that 7.71% of all deaths resulted from non-optimal temperatures, and cold exposure can be a serious threat [6]. Besides extreme weather, natural disasters such as war, tsunamis, and earthquakes add to these effects [7,8].

One of the most dangerous effects of accidental hypothermia is drowning in seawater, because seawater temperature is much lower than human body temperature. Additionally, seawater is more thermally conductive than air and offers more efficient convective heat transfer. Therefore, CBT of people immersed in water drops fast, which increases hypothermia and is life-threatening for individuals exposed to it. Seawater immersion hypothermia involves numerous pathophysiological phenomena, including high heat loss through conduction and convection, tissue hypoperfusion resulting from peripheral vasoconstriction, metabolic disturbances, and dysregulation of neuroendocrine systems [7,9]. Additionally, severe complications like skin and soft tissue wound injuries, wound infection, electrolyte imbalance, and more may happen [10,11].

Accidental hypothermia can be classified as mild hypothermia (CBT between 32 °C and 35 °C), moderate hypothermia (CBT between 28 °C and 32 °C), and serious hypothermia (CBT below 28 °C) [12]. There is evidence that sustained hypothermia strongly affects many organ systems, including the brain, heart, kidneys, immune system, and coagulation [13,14,15].

Current treatments tend to be passive or active external rewarming, for help when people are going through evacuation, but not for people who stay in seawater [16,17]. Furthermore, the sea has a large amount of dynamic and complex environments, making seafarers’ searches and rescues very difficult. Therefore, increasing the survival time of people trapped in seawaters to increase the chances of recovery is of high interest for research and practical purposes. In this context, further investigation of the underlying thermogenic activity of the body at low temperature, and the pharmacologically intervenable strategies to be applied in the field, is of great importance to help to improve the survival rate of seawater immersion hypothermia patients.

When exposed to cold, non-shivering thermogenesis mainly triggered by brown adipose tissue (BAT) is critical for mammals to resist hypothermia [18]. The thermogenic function of BAT mainly depends on uncoupling protein 1 (UCP1) in the inner mitochondrial membrane [19,20]. When active, UCP1 uncouples the mitochondrial respiratory chain from adenosine triphosphate (ATP) production, eliminating the proton gradient as heat. The sympathetic nervous system and downstream signaling pathways regulate this process [21,22]. Cold stimulation triggers sympathetic nerves and Norepinephrine (NE) release. NE binds to β3-adrenergic receptors (β3-AR) on BAT cell membranes to activate adenylate cyclase, increase intracellular cyclic adenosine monophosphate (cAMP), and stimulate protein kinase A (PKA) [23,24,25]. NE-(β3-AR)-cAMP-PKA signaling cascade activates lipolysis and upregulates UCP1 transcription, which forms one of the main molecular processes driving non-shivering thermogenesis. Further, the p38 mitogen-activated protein kinase (p38 MAPK) signaling pathway is found to play a key role in mediating cold- or adrenergic-induced thermogenesis of BAT [26,27]. When the p38 MAPK receptor is activated, it will phosphorylate and activate peroxisome proliferator-activated receptor-inactivator-1α (PGC1α), a core transcriptional coactivator that regulates mitochondrial biogenesis and function [28,29,30,31]. Activated PGC1α cooperates with nuclear receptors like peroxisome proliferator-activated receptors (PPARγ) to bind to the promoters of a thermogenic gene such as UCP1, which can greatly boost transcriptional expression [32,33,34,35,36]. Thus, the p38 MAPK/PGC1α/PPARγ signaling pathway is another upstream cascade that controls UCP1 expression and affects the thermogenic capacity of BAT.

There are many studies that reveal that traditional Chinese medicine (TCM) has unique therapeutic value to handle extreme and critical patients such as hypothermia, serious burns, heat stroke, and cerebral hemorrhage [37,38,39]. One of the classic TCM formulas, Shenfu Decoction (SFD), first documented in the Jisheng Xufang during the Song Dynasty, has a clinical application history of over 800 years. It is commonly used in the treatment of critical conditions in TCM, including yang collapse syndrome and syncope. It is composed only of *Panax ginseng* and *Aconitum carmichaelii* with commonly used ratios of 2:1, 1:1, and 1:2, and a clinical dosage range of 15–120 g [40,41,42]. *Panax ginseng* replenishes qi for the acquired constitution, and aconite strengthens yang for the innate constitution. When used together, the two herbs warm and tonify the heart and kidneys rapidly, recover pulse, and avoid collapse. Recent clinical and experimental results demonstrate that SFD can substantially increase the sensitivity to hypoxia, suppress excessive inflammation, and improve the haemodynamic parameters, which provide multi-target protective effects to hypoxaemia, septic shock, and chronic heart failure [43,44,45]. Thus far, many studies on seawater immersion hypothermia primarily focus on pathological mechanisms and rewarming strategies, with few studies investigating the potential therapeutic agents. Notably, preventive drug administration in order to prolong survival time and reduce mortality rate is exceptionally rare. Since extending survival time under cold seawater immersion significantly increases the likelihood of successful search, rescue, and subsequent medical evacuation, we innovatively investigated the effect of prophylactic SFD administration on survival time in rats subjected to seawater immersion. Our previous publication showed that SFD (1:1, 1.35 g/kg) extended survival time by approximately 6 h in rats immersed in 15 °C seawater [46]. In the present study, we further evaluated the effects of SFD with three common different *Panax ginseng* and *Aconitum carmichaelii* ratios (2:1, 1:1 and 1:2) on survival time. Additionally, the chemical and plasma-absorbed components of SFD are analyzed using an Ultra-Performance Liquid Chromatography-Quadrupole Time-of-Flight-Mass Spectrometry (UPLC-Q-TOF-MS) [47,48,49]. These absorbed components were then subjected to network pharmacology to predict targets and pathways for the anti-hypothermic effect of SFD (1:1) [50]. Mechanistic experiments on BAT thermogenesis have also been performed in order to find how SFD (1:1) upregulates UCP1 expression and activates related signaling channels. The novelty of this study lies in systematically evaluating the prophylactic effects of different SFD ratios on seawater immersion hypothermia for the first time and elucidating that SFD promotes BAT thermogenesis probably via upregulation of UCP1 through the p38 MAPK/PGC1α/PPARγ and NE-(β3-AR)-cAMP-PKA pathways. This has given a scientific motivation to optimize SFD formulation and to treat hypothermia caused by seawater immersion.

## 2. Results

### 2.1. Identification of the Chemical Constituents in SFD of Different Ratios

**Figure 1:**
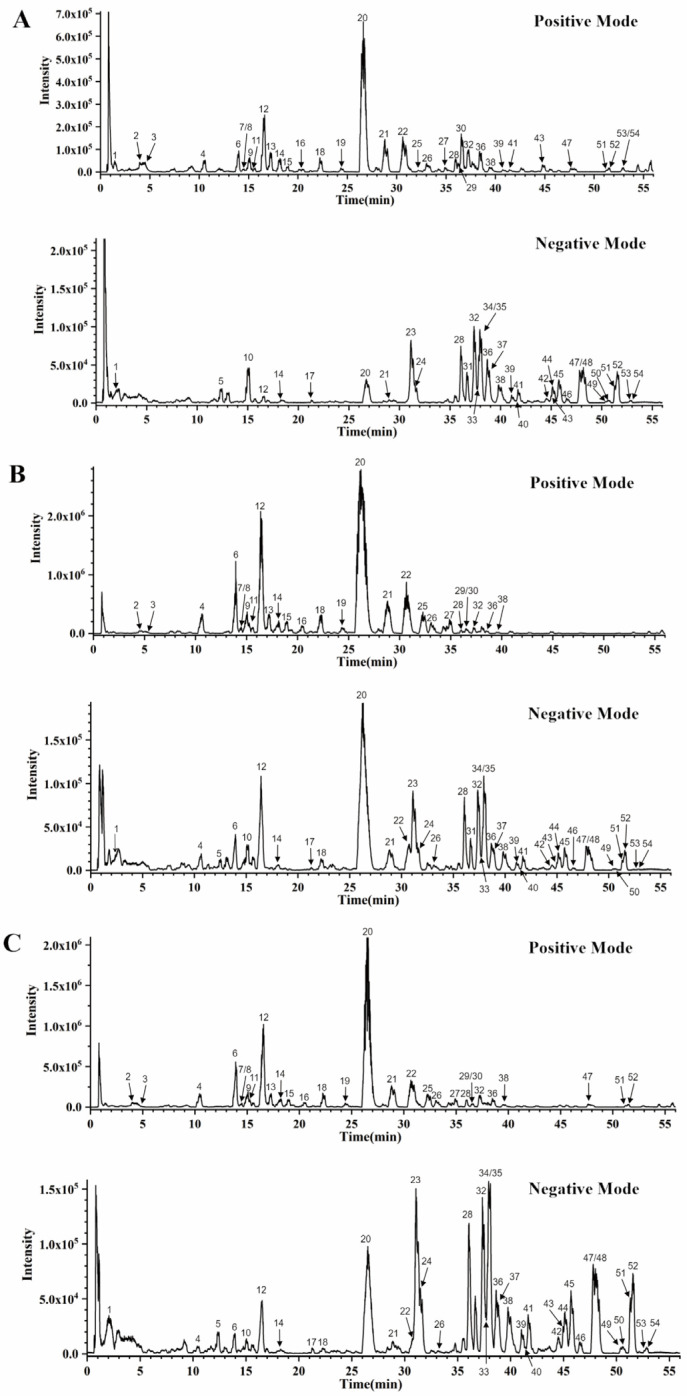
UPLC-Q-TOF-MS analysis of different proportions of SFD. (**A**) BPC in positive and negative ion modes of SFD (1:1). (**B**) BPC in positive and negative ion modes of SFD (1:2). (**C**) BPC in positive and negative ion modes of SFD (2:1). SFD: Shenfu Decoction; BPC: Base peak chromatogram; UPLC-Q-TOF-MS: Ultra-Performance Liquid Chromatography-Quadrupole Time-of-Flight-Mass Spectrometry.

Through a comprehensive component analysis of SFD at three different ratios—1:1, 1:2, and 2:1—a total of 54 common compounds were identified (Figure 1 and Table 1). Screening of the primary monomers of *Panax ginseng* and *Aconitum carmichaelii* in each ratio showed that ginsenoside Rg1, ginsenoside F1, benzoyl neoaconitine, aconitine, and benzoyl subaconitin are dominant for all three ratios, with certain monomers appearing at different ratios. From the content analysis, significant fluctuations of major ginsenosides and aconitine monoester alkaloids were seen for each ratio. The total proportion of ginsenosides for 1:1, 1:2, and 2:1 ratios was 25.30%, 3.45%, 11.93%, respectively. On the other hand, the total proportions of aconite monoester-type alkaloids were 39.70%, 55.53%, and 50.02%, respectively. Additionally, the overall proportions of the major toxic factors of aconitine (neoaconitine, aconitonine, subaconitinine) were 6.90%, 5.63%, and 5.86% across the three ratios. These results suggest that at different ratios, the percentage of primary monomer components in SFD is affected. Therefore, it is important to investigate the preventive effects of each ratio of SFD on rats experiencing hypothermia due to seawater immersion [51,52,53,54,55,56,57].

### 2.2. A Comparison of Different Proportions of SFD on Survival Times in Hypothermic Rats Immersed in Seawater

**Figure 2:**
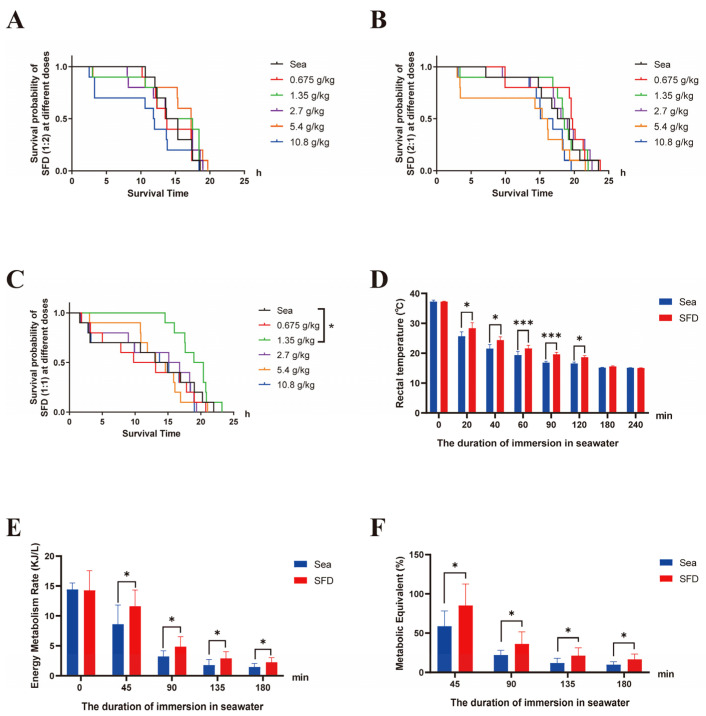
Survival analysis and energy metabolism analysis of SFD with different proportions. (**A**) Effects of different dosages of SFD (1:2) on survival duration in rats with hypothermia induced by seawater immersion (n = 10). (**B**) Effects of different dosages of SFD (2:1) on survival duration in rats with hypothermia induced by seawater immersion (n = 10). (**C**) Effects of different dosages of SFD (1:1) on survival duration in rats with hypothermia induced by seawater immersion (n = 10). (**D**) Bar chart illustrating the effect of SFD (1:1) at dosages of 1.35 g/kg on CBT in a rat model of seawater-immersion-induced hypothermia (n = 10). (**E**) The impact of SFD (1:1) at dosages of 1.35 g/kg on energy metabolism in rats with hypothermia induced by seawater immersion (n = 9). (**F**) The impact of SFD (1:1) at dosages of 1.35 g/kg on metabolic equivalent in rats with hypothermia induced by seawater immersion (n = 9). Quantitative data are expressed as mean ± SD. Sea: a model group subjected to hypothermia induction via seawater immersion immediately after gavage with distilled water; SFD: a Shenfu Decoction group subjected to hypothermia induction via seawater immersion immediately after gavage with Shenfu Decoction. * *p* < 0.05, *** *p* < 0.001, versus Sea group.

Survival analysis showed that the relative survival time of rats under hypothermic seawater immersion decreased considerably. The mean survival time of the seawater-immersed model group (Sea group) was 12.45 ± 7.60 h (Figure 2A–C). When pretreatment with SFD (1:1) using oral gavage, the mean survival times increased to 19.03 ± 2.60 h (*p* < 0.05). The other dose groups of SFD (1:1) were not statistically different from the Sea group, indicating that the mean survival time during hypothermia due to seawater submergence was not dose dependent. Furthermore, with herbal proportion conditions of 1:2 and 2:1, none of them had statistical differences with the Sea group. Since SFD (1:1, 1.35 g/kg) proved to be the most effective among the three common tested formulations in extending survival time in a rat model of seawater-immersion-induced hypothermia, this regimen was used for all subsequent pharmacodynamic mechanism studies.

### 2.3. The Effect of SFD on the CBT of Hypothermia Rats Immersed in Seawater

CBT is an important indicator for body thermogenesis. In this research, we observed changes in CBT in several groups of rats being subject to a hypothermia model from seawater immersion using a precise rectal thermometer. The outcome showed no significant difference between the seawater-immersed model group (Sea group) and the SFD group at baseline. However, the CBT of the SFD group exceeded that of the Sea group at 20 min (*p* < 0.05). At 20 min, 40 min, 60 min, 90 min, and 120 min, the mean rectal temperature of the SFD group was 28.36 ± 1.84, 24.42 ± 1.10, 21.60 ± 1.04, 19.62 ± 0.70, and 18.66 ± 0.53 °C, respectively. The difference between the SFD and Sea group is also significant at all these times, so pretreatment with SFD (1:1) at 1.35 g/kg could increase rectal temperatures of rats at around 2 °C during 20–120 min of seawater immersion. Overall, SFD greatly slowed down the decrease in CBT in hypothermia-induced seawater immersion rats (Figure 2D).

### 2.4. The Effect of SFD on Energy Metabolism in Hypothermic Rats Immersed in Seawater

Continuous respiratory metabolism monitoring showed that the energy metabolic rate of all rats decreased significantly with hypothermic seawater immersion, especially during the first 45 min, consistent with core temperature changes. No difference was observed between the Sea group and the SFD group in resting metabolic rate. In four measurements, both energy metabolic and metabolic equivalent of the SFD group were significantly higher than those of the Sea group, with statistical significance (Figure 2E,F). This indicates that SFD is capable of greatly upregulating energy metabolism-relevant indicators for hypothermia of the rats with seawater submerging, and provides direct evidence of its thermogenic origin.

### 2.5. Characterization of Plasma-Absorbed Compounds of SFD

**Figure 3:**
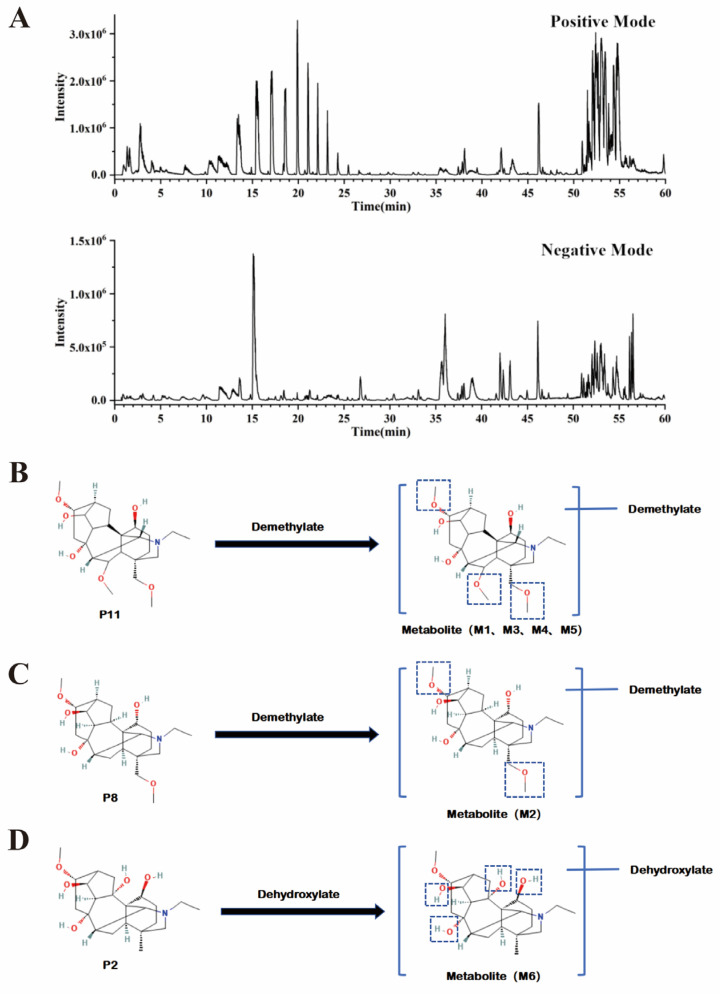
UPLC-Q-TOF-MS analysis of plasma-absorbed compounds in SFD (1:1, 1.35 g/kg). (**A**) Base peak chromatogram in positive and negative ion modes. (**B**) The metabolic pathways of metabolites M1, M3, M4, and M5. (**C**) The metabolic pathways of metabolite M2. (**D**) The metabolic pathways of metabolite M6. UPLC-Q-TOF-MS: Ultra-Performance Liquid Chromatography-Quadrupole Time-of-Flight-Mass Spectrometry; SFD: Shenfu Decoction; The dashed box delineates a moiety susceptible to demethylation or dehydroxylation.

After 2 h of intragastric administration of SFD at dosages of 1.35 g/kg, 34 chemical constituents were found in the serum of rats subject to seawater immersion, including 28 prototypes and 6 metabolites. The prototype constituents (P1–P28) and metabolites (M1–M6) were identified by their retention times, molecular weights, molecular formulas, and MS/MS fragmentation patterns. The final constituents were 18 alkaloids and 9 ginsenosides in addition to Eucomic acid (Table 2). In the metabolites studied, 6 were alkaloids. The main pathway for the alkaloidal metabolites was demethylation (M1–M5) and dehydroxylation (M6) (Figure 3 and Table 3).

### 2.6. Network Pharmacology Study

#### 2.6.1. Target Identification of SFD’s Main Components and Hypothermia

**Figure 4:**
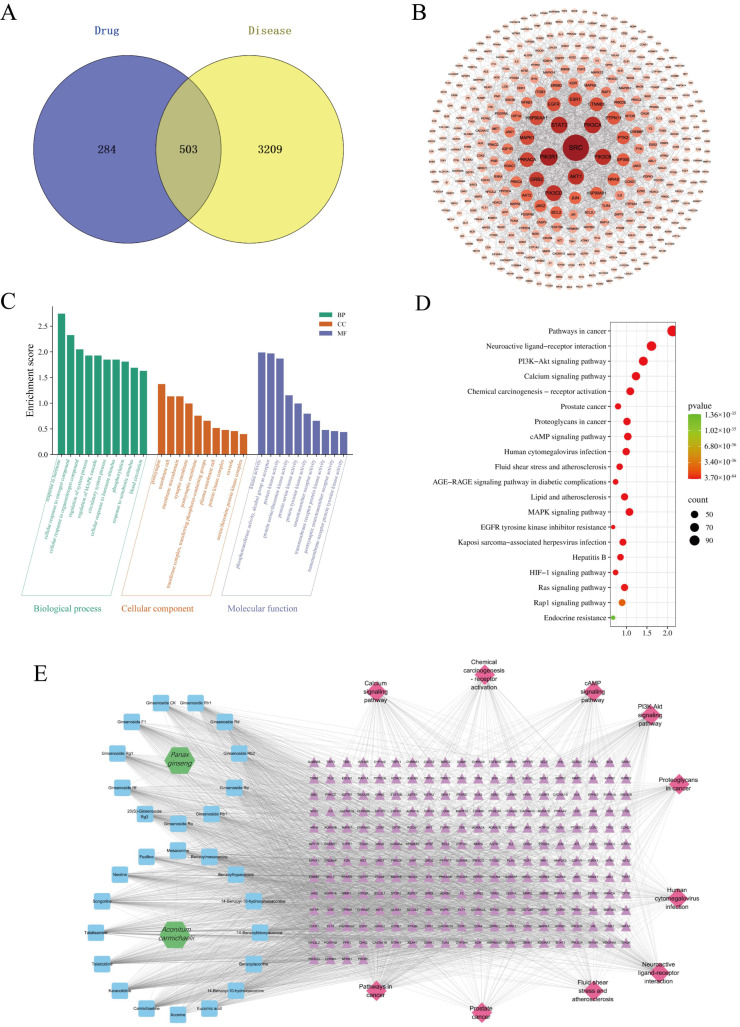
Network pharmacology analysis of SFD (1:1, 1.35g/kg) in a rat model of seawater-immersion-induced hypothermia. (**A**) Venn diagram of the targets of SFD and hypothermia. (**B**) The PPI network of SFD and hypothermia core targets. (**C**) GO pathway enrichment analysis of core targets. (**D**) KEGG pathway enrichment analysis of core targets. (**E**) Network of drug components—potential targets—regulatory pathway. SFD: Shenfu Decoction; PPI: protein–protein interaction; GO: Gene Ontology; KEGG: Kyoto Encyclopedia of Genes and Genomes.

Based on the findings from the characterization of plasma-absorbed compounds, we discovered 28 bioavailable candidate compounds in SFD using UPLC-Q-TOF-MS. From these compounds, we extracted molecular information of the corresponding pharmacologically active ingredients from the PubChem database (pubchem.ncbi.nlm.nih.gov). Potential drug targets were predicted online using SuperPred, SwissTargetPrediction, and PharmMapper. A total of 787 drug-related targets were obtained. Additionally, disease-related targets were primarily sourced from the GeneCards database. A search was conducted using 24 keywords, which yielded a total of 3712 targets associated with this condition. Venn analysis revealed 503 potential targets of action between drug components and hypothermia (Figure 4A).

#### 2.6.2. Protein–Protein Interaction (PPI) Analysis of Core Targets for SFD in Improving Hypothermia

As shown in Figure 4B, the network comprises a total of 397 nodes and 1524 edges, with an average node degree of 7.678. Among these, 124 nodes exhibit a degree higher than the average, with SRC having the highest degree (degree = 52), followed by PIK3R1, PIK3CA, STAT3, and AKT1. The CytoHubba plugin further identified PTPN11, PIK3R1, and PIK3CA as key nodes within the network.

#### 2.6.3. Enrichment Analysis of Gene Ontology (GO) for Potential Core Targets

GO enrichment analysis revealed that SFD alleviates seawater-immersion-induced hypothermia primarily by modulating responses to xenobiotic stimuli and regulating the mitogen-activated protein kinase (MAPK) cascade. Additional mechanisms include the regulation of cellular responses to nitrogen compounds and hormonal stimuli, as well as the modulation of neurotransmitter receptor activity (Figure 4C).

#### 2.6.4. Enrichment Analysis of KEGG Pathways for Potential Core Targets

The KEGG enrichment analysis of the 503 potential targets was further conducted. First, the potential targets were imported into the Metascape database for KEGG enrichment analysis, with *p* < 0.05 set as the screening threshold [58]. Subsequently, the ggplot2 package was utilized to visualize the results in the form of bubble charts, sorted by their respective *p*-values. The analysis results are presented in Figure 4D. The SFD-mediated improvement of hypothermia induced by seawater immersion primarily involves pathways such as the cAMP signaling pathway, the MAPK signaling pathway, the Rap1 signaling pathway, and so on [59]. Notably, the cAMP signaling pathway is a well-established upstream regulatory axis for thermogenesis, which classically modulates the expression of UCP1 in BAT [60,61,62]. This suggests that SFD may exert its therapeutic effects on hypothermia, potentially through activating thermogenic programs.

#### 2.6.5. “Drug Components—Potential Targets—Regulatory Pathway” Network

After collating drug targets, pathways, and chemical constituents, we constructed an integrated regulatory network comprising 283 nodes and 2589 edges (mean degree = 18.297). Targets are shown as central purple nodes, whereas constituents and pathways are represented by blue and pink nodes, respectively (Figure 4E). Topological analysis identified Carmichaeline, Karacolidine, Talatizidine, Talatisamine, 20(S)-Ginsenoside Rg3, and Songorine as the most highly connected constituents, implicating them as putative core bioactives through which SFD mitigates seawater-immersion-induced hypothermia. The ten most influential targets—PIK3R1, NFKB1, PDGFRA, CHUK, PIK3CD, CHRM1, MTOR, and PIK3CA—emerged as candidate intervention points for SFD.

### 2.7. The Effect of SFD on the Core Thermogenic Target UCP1 in BAT of Hypothermia Rats

**Figure 5:**
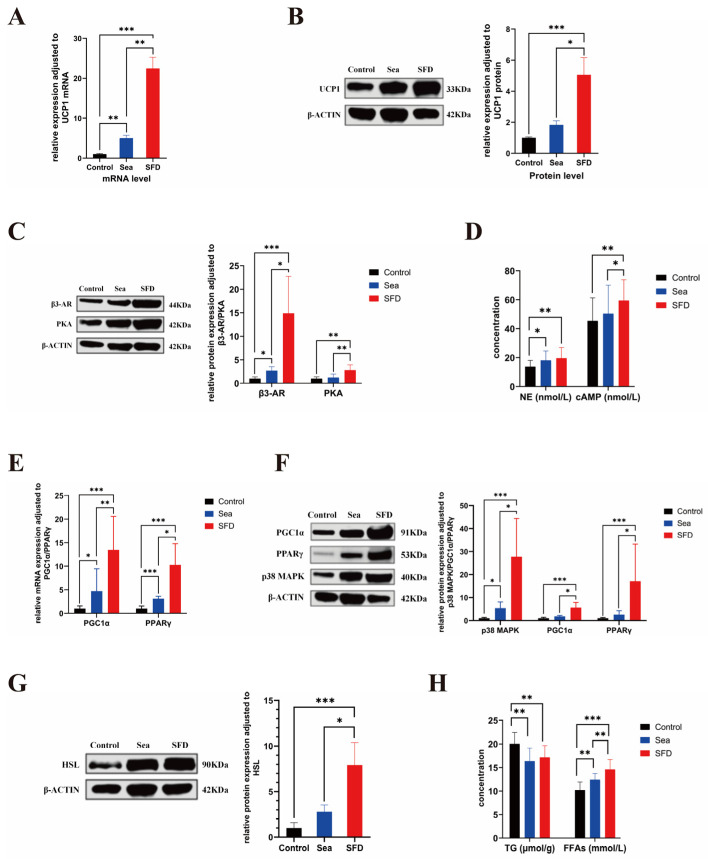
SFD alleviates seawater-immersion-induced hypothermia in rats through modulation of BAT’s relevant targets and pathways. (**A**) Relative mRNA expression of UCP1 in BAT (n = 6). (**B**) Western blot (WB) analysis of UCP1 in BAT (n = 8). (**C**) WB analysis of β3-AR and PKA in BAT (n = 9). (**D**) ELISA analysis of NE and cAMP in BAT. (n = 11). (**E**) Relative mRNA expression of PGC1α and PPARγ in BAT (n = 6). (**F**) WB analysis of PGC1α, PPARγ and p38 MAPK in BAT (n = 8). (**G**) WB analysis of HSL in BAT (n = 9). (**H**) ELISA analysis of TG and FFAs in BAT (n = 12). Quantitative data are expressed as mean ± SD. Control: a control group without any treatment; Sea: a model group subjected to hypothermia induction via seawater immersion immediately after gavage with distilled water; SFD: a Shenfu Decoction group subjected to hypothermia induction via seawater immersion immediately after gavage with Shenfu Decoction (1:1, 1.35g/kg); BAT: Brown adipose tissue; UCP1: Uncoupling Protein 1; β3-AR: Beta-3 Adrenergic Receptor; PKA: Protein Kinase A; NE: Norepinephrine; cAMP: Cyclic Adenosine Monophosphate; PGC1α: Peroxisome Proliferator-Activated Receptor Gamma Coactivator 1-Alpha; PPARγ: Peroxisome Proliferator-Activated Receptor Gamma; p38 MAPK: p38 Mitogen-activated protein kinase; HSL: hormone-sensitive lipase; TG: triglycerides; FFAs: free fatty acids (* *p* < 0.05, ** *p* < 0.01, *** *p* < 0.001).

UCP1 is currently recognized as one of the most important proteins regulating thermogenesis in BAT. Given that the present study showed that SFD significantly increased the rectal temperature of rats with seawater-immersion-induced hypothermia, we further analyzed the effects of SFD on UCP1 expression at both the transcriptional and translational levels. The reverse transcription-polymerase chain reaction (RT-PCR) analysis revealed that, compared to the control group, UCP1 mRNA expression was elevated in the seawater-immersed model group (Sea group) (*p* < 0.01), and was further significantly increased by SFD treatment (*p* < 0.001) (Figure 5A). Similarly, WB analysis showed that UCP1 protein expression was moderately induced in the Sea group compared to the control group, while SFD administration led to a marked upregulation of UCP1 protein levels (*p* < 0.05) (Figure 5B). These results suggest that low temperatures can partially induce the UCP1 expression, and SFD significantly enhances the UCP1 protein production in hypothermic rats. In all cases, these findings indicate that SFD is beneficial for the UCP1 expression, which is a key thermogen.

### 2.8. The Effect of SFD on Core Heat-Producing Target UCP1 and Its Upstream Molecules in the BAT of Hypothermic Rats

RT-PCR results showed that, compared to the control group, the standardized fold changes in the Sea and SFD groups are 5.00 ± 2.32 and 22.46 ± 8.93, respectively (*p* < 0.05), suggesting hypothermic seawater immersion led to UCP1 gene transcription, and SFD increased UCP1 transcription levels significantly (Figure 5A). Western blot studies showed that, compared to the control group, standardized fold changes in greyscale values for the Sea and SFD groups are 1.83 ± 0.82 and 5.05 ± 3.51, respectively (*p* < 0.05). All these results suggest that SFD supports the expression of UCP1, the main thermogenic target of BAT of rats during seawater hypothermia (Figure 5B).

To further investigate the thermoregulatory effects of SFD on BAT in hypothermic rats, we analyzed the upstream signaling pathways. The results demonstrated that the expression of β3-AR protein in the Sea group was much higher than that of the control group, although the level of PKA was slightly increased, which did not reach statistical significance. After intragastric administration with SFD, the expression level of β3-AR and PKA protein also increased (*p* < 0.05) (Figure 5C), suggesting that SFD could enhance the expression levels of key receptors in this signaling pathway. For signaling molecule concentrations, the NE level in the Sea group is substantially higher than in the control sample (*p* < 0.05). Although cAMP levels increased slightly in the Sea group compared to the control group, they were not significant. The cAMP concentrations of the SFD group increased compared with the control (*p* < 0.01) and Sea group (*p* < 0.05), and NE levels remained unchanged (Figure 5D). We conclude that SFD increases cAMP levels and helps downstream PKA signal transduction. In summary, SFD probably activates the NE-(β3-AR)-cAMP-PKA pathway, promotes the expression of β3-AR and PKA proteins, and enhances thermogenic regulation in BAT under low temperature.

Furthermore, we also investigated the effect of SFD on the expression of other key signaling molecules in the transcriptional cascade reaction. At the mRNA level, protein levels of PGC1α and PPARγ increased significantly with SFD (*p* < 0.001) compared to those of the Sea group (*p* < 0.05) (Figure 5E). On the protein level, the protein levels for p38 MAPK, PGC1α, and PPARγ in the SFD group were significantly higher than for the control group (*p* < 0.001) and the Sea group (*p* < 0.05), indicating a general upregulation trend (Figure 5F). These results suggest that SFD can facilitate transcription activation by activating the p38 MAPK/PGC1α/PPARγ pathway.

Another surprising improvement in lipid metabolism was achieved by SFD using WB analysis. In the SFD group, hormone-sensitive lipase (HSL) expression was larger than that of the control (*p* < 0.001) (Figure 5G). Metabolites also showed that the concentration of free fatty acids (FFAs) in the SFD group significantly exceeded that of the Sea group (*p* < 0.01) and that the triglyceride (TG) concentration was unchanged. This suggests that SFD tends to release FFAs by increasing HSL-mediated lipolysis (Figure 5H). Finally, SFD may synergistically activate the p38 MAPK/PGC1α/PPARγ transcriptional pathway and boost HSL-mediated lipolysis, thereby increasing the expression of downstream thermogenic protein UCP1 and enhancing the thermogenic capacity of BAT at low temperatures.

### 2.9. The Effects of SFD on BAT Morphology in Hypothermic Rats with Seawater Immersion

**Figure 6:**
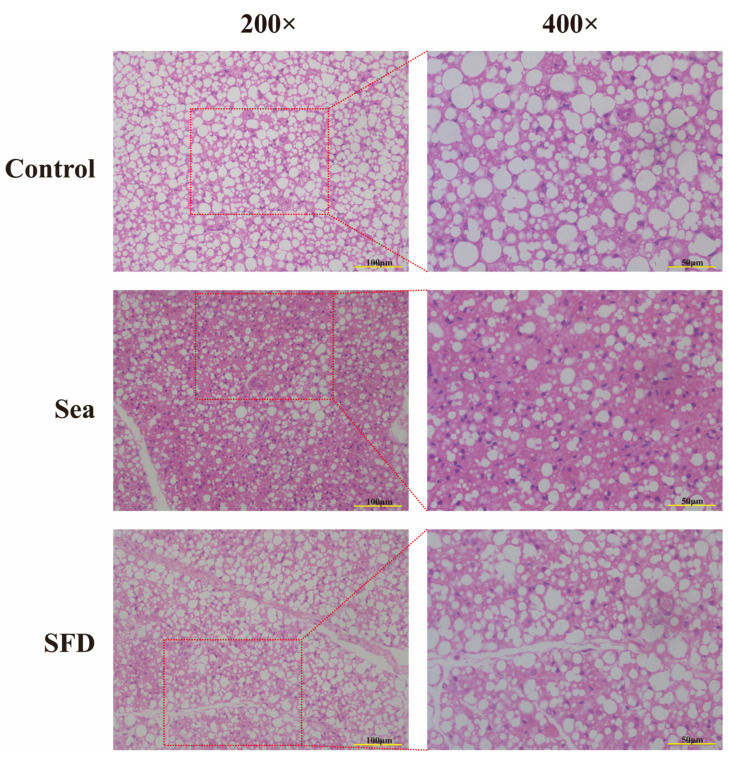
H&E staining analysis of the morphology of Brown adipose tissue in different groups of rats. Control: a control group without any treatment; Sea: A model group subjected to hypothermia induction via seawater immersion immediately after gavage with distilled water; SFD: A Shenfu Decoction group subjected to hypothermia induction via seawater immersion immediately after gavage with Shenfu Decoction (1:1, 1.35 g/kg); H&E: Hematoxylin and Eosin stainin.

Hematoxylin and Eosin stainin (H&E) staining pathological sections showed that brown adipocytes of the control, Sea, and SFD groups mainly differ in lipid droplet sizes and distribution. Brown adipocytes in the control group generally carried large lipid droplets, and the overall distribution was relatively dense. In the Sea group, the number of large lipid particles dropped significantly, and small lipid particles decreased as well as overall lipid particle numbers, smaller droplet size, and looser distribution. In the SFD group, the number of cytoplasmic lipid droplets was lower than that in the control group but significantly higher than that in the Sea group, with droplet size also intermediate between the two groups. In addition, small and large lipid quantities were mixed and distributed, and small droplets dominated (Figure 6).

## 3. Discussion

Seawater immersion hypothermia is a special kind of accidental hypothermia, due to the much higher thermal conductivity of seawater than air, leading to a more rapid decline in CBT [63]. Cold seawater immersion is a typical form of cold stress. As CBT drops, patients develop increasingly weak vital signs, including cold extremities, shallow and slow breathing, bradycardia, and impaired consciousness. Notably, the cause and clinical presentation of seawater-immersion-induced hypothermia closely resemble syncope in TCM, especially the type caused by yang qi deficiency due to pathogenic cold. The treatment aims to revive yang, restore collapse, stabilize qi, and reinforce the body’s recovery. Therefore, we used the traditional Chinese herbal decoction SFD as an intervention for seawater-immersion-induced hypothermia, particularly when given before cold seawater exposure, to prolong survival time and lower mortality, thus addressing the research gap on preventive pharmacotherapy for this condition.

SFD is composed of two herbal medicines, *Panax ginseng* and *Aconitum carmichaelii*. Throughout its long history of clinical application, three different ratios have been commonly employed by TCM practitioners. To systematically identify the most effective ratio and dosage of SFD, we prepared three different formulations using the same raw herbs and following an identical decoction protocol. Survival analysis revealed that only the 1:1 ratio of SFD, previously administered at a dosage of 1.35 g/kg, significantly prolonged the survival time of rats subjected to cold seawater immersion. From the perspective of herbal compatibility, TCM theory holds that the 1:1 ratio of SFD enhances therapeutic efficacy while mitigating toxicity. The UPLC-Q-TOF-MS analysis also demonstrated that compared with the other two ratios, the 1:1 formulation contained higher levels of ginsenosides and lower levels of aconitine, which is the most potent toxic component in *Aconitum carmichaelii*. Furthermore, some pharmacological studies provide additional insights into the benefits of the SFD from alternative perspectives. For instance, *Panax ginseng* may decrease the metabolism, excretion, or plasma clearance rate of the monoterpenoid alkaloid active components from *Aconitum carmichaelii*, thereby enhancing efficacy [64]. Additionally, ginsenoside Rg1 is found to be able to absorb benzoic aconitine and also metabolize aconitine [65]. Determining the optimal dosage of prophylactic SFD for prolonging survival time in rats following cold seawater immersion is of critical importance. Survival curve analysis did not reveal a classical dose-dependent trend; instead, only the 1:1 ratio at a dosage of 1.35 g/kg was found to be effective. This finding may be attributable to the following two reasons. First, in our initial investigation of the effects of various SFD ratios and dosages on mortality in a rat model of seawater-immersion-induced hypothermia, we tested five concentrations ranging from 0.675 to 10.8 g/kg (equivalent to the clinical dosage range of 7.5–120 g), with a twofold incremental increase, to comprehensively screen for effective concentrations. Second, the absorption, distribution, metabolism, and excretion of drugs, including TCMs, are closely related to the physiological state of the organism. Numerous studies have demonstrated that the pharmacokinetics of various drugs, such as anesthetics and antibiotics, are significantly altered under hypothermic conditions [66,67], suggesting that precise dose control of these agents is warranted when treating patients with clinical hypothermia. Therefore, it is reasonable to postulate that, in the context of seawater-immersion-induced hypothermia, TCMs may also exert their therapeutic effects within a limited or even narrow concentration range.

In response to cold stress, the body preserves core temperature by minimizing heat dissipation (via cutaneous vasoconstriction) while enhancing thermogenesis. However, as cold exposure intensifies, the progressive failure of thermoregulatory homeostasis precipitates the onset of hypothermia. CBT is the simplest indicator of hypothermia from seawater exposure. Common measures of core temperature in rats are abdominal, rectal, and thoracic temperatures. For these, abdominal and thoracic temperatures are the most accurate measures of core temperature, but with invasive procedures such as implanted recorders of temperature in the abdomen. Here, rectal temperature is used to track core temperature to minimize the effect of invasive procedures. To minimize measurement errors during the repeated assessment of rectal temperature in rats, all measurements were performed by a single experienced experimenter following a standardized protocol. The results revealed that SFD dramatically increased CBT in a rat undergoing seawater immersion hypothermia. It is observed that even a slight drop in CBT could lead to a nonlinear increase in the risk of ventricular fibrillation and neurological decline [68,69,70]. Therefore, the 2–3 °C gain in body temperature of SFD theoretically could help to shorten the onset of life-threatening arrhythmias and to delay the progression of neurological dysfunction, which is crucial for on-site first aid and later medical rescue. Respiratory metabolism measurement reflects the basal metabolic state and is closely associated with thermogenesis. Our experimental results of the present study showed that, consistent with the CBT findings, SFD also attenuated the decrease in respiratory metabolic rate induced by cold seawater immersion, thereby further confirming its thermogenic efficacy. Regarding the mechanisms, previous studies have investigated the thermoregulatory effect of *Panax ginseng* and aconite in low-temperature environments: for example, Hong et al. have found that *Panax ginseng* affects the body temperature of mice in normal, hyperthermic, and hypothermic conditions, revealing that *Panax ginseng*’s thermoregulatory effect was more pronounced under low-temperature conditions. The results suggest that under low-temperature conditions, mice consuming *Panax ginseng* can recover their body temperature quickly [71]. It is also found that the low-temperature rats with intermittent treatment of *Panax ginseng*, which significantly enhances their average and maximum CBT, may be related to increased energy consumption from the sympathetic nervous system and thyroid hormones [72,73]. Another important influence of aconite on hypothermia is also important: it can mitigate the reduction in CBT when mice suffer from chronic cold exposure, possibly by increasing the expression of UCP1 in BAT [74]. The fresh extract of aconite also seems to help to eliminate hypothermic symptoms from the prolonged intermittent cold exposure of rats, with infrared thermal images showing thick hot zones around brown adipose [75]. Another study found that fresh extract of aconite has increased CBT and resistance to cold injury by altering gut microbiota and bile acid metabolism [76].

Network pharmacology is currently one of the most commonly used approaches for predicting the mechanisms of action of traditional Chinese herbs and their formulas. Conventional network pharmacology analyses are typically based on data from public TCM databases. However, the *in vitro* bioactive components of TCMs are influenced by multiple factors, including herbal quality, decoction method, and herbal compatibility. Moreover, the actual absorbed components *in vivo* differ substantially from the *in vitro* constituents, owing to factors such as administration route, dosage, and disease model. Notably, since hypothermia may affect the absorption, metabolism, distribution, and excretion of drugs, including TCM formulas, we did not use the 54 *in vitro* components of SFD for the network pharmacology analysis. Instead, we utilized the 28 absorbed constituents of SFD identified in rat serum by UPLC-Q-TOF-MS (Figure 4A), with the aim of obtaining a more reasonable and realistic prediction of the potential mechanisms of action. GO functional enrichment shows that SFDs’ targets may have biological influences, such as response to xenobiotic stimulus, regulation of the MAPK pathway, and hormone regulation. Consistent with existing literature, the p38 MAPK/PGC1α/PPARγ axis serves as a branch of the MAPK pathway and a key mediator of UCP1 transcriptional activation. Upon stimulation by cAMP/PKA or other upstream signals [77], p38 MAPK phosphorylates and activates PGC1α. Subsequently, activated PGC1α co-activates PPARγ, driving the expression of UCP1 and other mitochondrial oxidative genes in BAT. This regulatory linkage is further substantiated by key targets identified in our protein–protein interaction analysis and “drug-target-pathway” networks, specifically STAT3 and MTOR (Figure 4B,E). While STAT3 is established to regulate sympathetic nerve activity and thermogenesis [78,79,80], MTOR signaling can intersect with PGC1α/PPARγ activity under specific metabolic stress conditions [80,81]. Network pharmacology analysis predicted the cAMP and MAPK signaling pathways. However, both pathways serve as core signal transduction hubs that participate in the regulation of diverse physiological functions and pathological processes. Therefore, the precise molecular mechanisms by which SFD prolongs survival time in rats with seawater-immersion-induced hypothermia cannot be inferred solely from network pharmacology results. Instead, comprehensive analysis and experimental validation are required, integrating knowledge of the regulatory mechanisms of cold stress, the key pathological features of hypothermia, and the established pharmacological actions of *Panax ginseng* and *Aconitum carmichaelii*. For example, ginsenosides (e.g., Rg1, Rb1, and Rg3) in cold adaptation are shown to promote mitochondrial biogenesis and white adipose tissue browning through activation of PGC1α/PPARγ [82,83,84], reduce cold-induced organ damage by anti-inflammatory actions, such as suppressing the NF-κB pathway, and enhance antioxidative effects, such as activation of Nrf2/HO-1 pathways [85,86]. Active components from *Aconitum carmichaelii*, as well as total *Panax ginseng* extract, can enhance thermogenesis in hypothermic animals due to cold stress by further upregulating UCP1 levels in BATs, and a representative of *Aconitum carmichaelii*, fuziline, is shown to directly activate BAT thermogenesis via the NE-(β3-AR)-cAMP-PKA pathway [74,87,88]. Based on the above considerations, we propose that the ability of SFD to alleviate the specific cold stress injury induced by cold seawater immersion and to delay the progression of hypothermia is likely related to its activation of the NE-(β3-AR)-cAMP-PKA and p38 MAPK/PGC1α/PPARγ signaling pathways, which in turn upregulate UCP1 expression and enhance thermogenesis in BAT.

H&E staining of BAT revealed that, as a result of hypothermic seawater exposure, lipid droplet size and number were dramatically reduced in rat BAT. SFD treatment could increase the lipid droplet volume and restore the lipid and droplet number. Interestingly, while SFD treatment resulted in larger lipid droplets compared to the Sea group, this morphological change does not contradict the enhanced thermogenesis observed. On the contrary, it reflects an efficient metabolic state: under acute cold stress (Sea group), BAT undergoes rapid lipolysis to fuel thermogenesis, leading to shrunken lipid droplets. In contrast, SFD intervention likely upregulates mitochondrial function and UCP1 expression, thereby enhancing the efficiency of fatty acid oxidation. This allows sufficient heat production without necessitating the exhaustive consumption of lipid stores, thus preserving relatively larger and more intact lipid droplet morphology in the SFD group [22,89,90,91]. These results further suggested that SFD causes morphological changes in BAT and promotes thermogenesis. UCP1 is a key protein in BAT. WB and PCR data showed that UCP1 expression was increased both at the translational and transcriptional levels, suggesting that SFD promotes UCP1 expression by upregulating its transcription upstream. The bioinformatic findings and known pharmacological responses of ginsenosides and aconitum alkaloids indicate that thermogenesis-related p38 MAPK/PGC1α/PPARγ and NE-(β3-AR)-cAMP-PKA signaling pathways are likely its upstream pathways.

**Figure 7:**
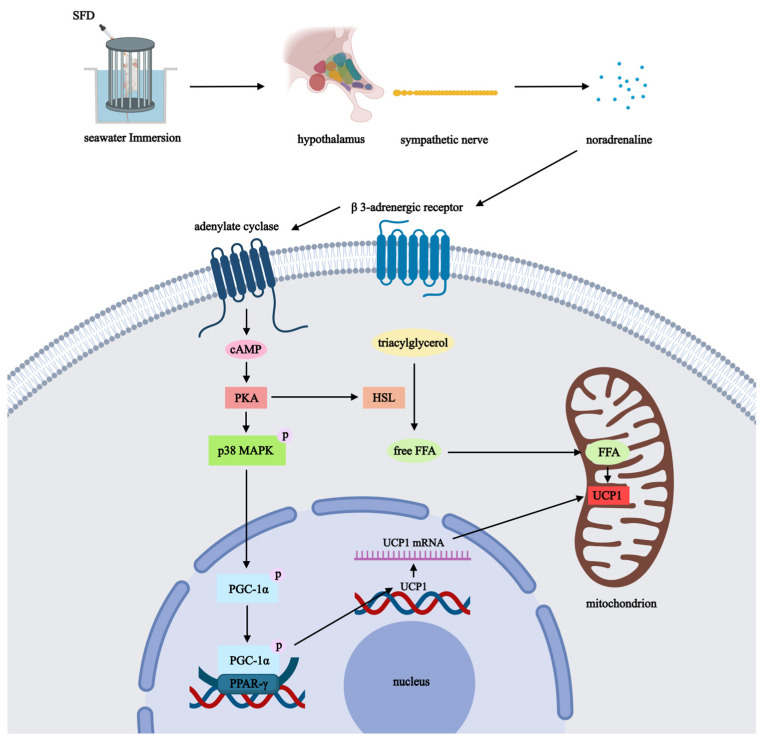
Hypothetical schematic illustration of the molecular mechanism underlying SFD-mediated modulation of thermogenic signaling pathways in rats with seawater-immersion-induced hypothermia. UCP1: Uncoupling Protein 1; PKA: Protein Kinase A; cAMP: Cyclic Adenosine Monophosphate; PGC1α: Peroxisome Proliferator-Activated Receptor Gamma Coactivator 1-Alpha; PPARγ: Peroxisome Proliferator-Activated Receptor Gamma; p38 MAPK: p38 Mitogen-activated protein kinase; HSL: hormone-sensitive lipase; SFD: Shenfu Decoction.

For the regulation of the NE-(β3-AR)-cAMP-PKA axis. When the NE level is sufficiently high due to prolonged sympathetic nervous system activation, β3-AR (abundantly expressed in brown fat) could both stimulate and inhibit G protein-coupled receptors, which maintain the steady adrenergic signal and enhance lipid oxidation and metabolic activity, such as lipid consumption and glucose uptake. The cAMP-PKA signaling pathway, mediated by these receptors, is widely believed to be the upstream mechanism for mitochondrial respiration and adaptive thermogenesis [24,92]. As activated by PKA, HSL is able to hydrolyse TG into FFAs in BAT: FFAs are not only direct energy substrates for β-oxidation but also bind to UCP1 to regulate protein conformation and functional expression [93,94]. In addition, PKA also activates downstream signaling molecules such as p38 MAPK and then modifies PGC1α by phosphorylation and deacetylation, which is key for the binding of nuclear receptors and respiratory chain operation. PGC1α, as coupled to PPARγ, binds to DNA response elements and allows transcriptional activation of thermogenic genes (such as UCP1) and enhances the replication and transcription of mitochondrial oxidative phosphorylation and thermogenesis, and finally positively impacts mitochondrial function and energy metabolism (Figure 7) [95,96,97]. However, it is important to note here that our evidence is primarily correlative. We investigated fluctuations in overall protein level expression of key proteins but did not show specific information on the phosphorylation status of kinases PKA and p38 MAPK. Moreover, some of the mechanisms underlying the onset of seawater immersion hypothermia are hormonal, the metabolism of energy substrates, cardiac dysfunction, and inflammatory response. Whether SFD also exerts its effects through these mechanisms awaits further in-depth investigation.

## 4. Materials and Methods

### 4.1. Chemicals and Materials

Artificial sea salt was acquired from Hai Zhi Yan Technology Co., Ltd. (Qingdao, China). Commercial ELISA kits for serum NE and cAMP were from Shanghai Langdun Biotechnology Co., Ltd. (Shanghai, China). Assay kits for FFAs and TG were from Nanjing Jiancheng Bioengineering Institute (Nanjing, China). The p38 MAPK, PKA, and β-actin antibodies were from Cell Signaling Technology (Danvers, MA, USA). The PPARγ, β3-AR and HSL antibodies were from Abcam (Cambridge, UK). The PGC1α and UCP1 antibodies were from Aibotech Biotechnology Co., Ltd. (Wuhan, China). Primer pairs for UCP1, PPARγ, PGC1α, and β-actin mRNA were from Shanghai Guantai Biotechnology Co., Ltd. (Shanghai, China). *Panax ginseng* (Chinese Red Ginseng, 2022080007) and *Aconitum carmichaelii* (2022093) were procured from Lei Yun Shang TCM Co., Ltd. (Shanghai, China). Experts in TCM identification from the Naval Medical University identified the plant materials. All samples and voucher specimens are stored in the drug warehouse of the Department of TCM, Naval Medical University.

### 4.2. Preparation of the SFD and Test Solution

Three types of herbs were prepared for the 1:1, 1:2, and 2:1 (g/g) *Panax ginseng* to *Aconitum carmichaelii* and soaked in 10 times the volume of distilled water for 1 h, boiled three times for 2 h, or distilled to 1.08 g/mL and stored at −20 °C to carry out an *in vitro* test. Transfer 1 mL of each sample into 10 mL centrifuge tubes, add 20% methanol, mix thoroughly, and centrifuge at 12,000 rpm for 5 min. Filter through a 0.22 µm nylon membrane for UPLC-Q-TOF-MS [98]. To prepare drug-containing serum test sample solutions, the serum sample was combined with methanol (mass spectrometry grade) at a ratio of one part serum to three parts methanol to facilitate protein precipitation. The mixture was subjected to vortex mixing for 5 min, followed by a 20 min incubation at 4 °C. Subsequently, the sample underwent centrifugation at 12,000 rpm for 15 min [59]. The supernatant was then collected, concentrated, dried, and stored at −80 °C prior to analysis, the dried residue was reconstituted in 100 µL of 50% methanol, vortex-mixed for 3 min, and centrifuged again at 12,000 rpm for 15 min. The final test sample was obtained from the resulting supernatant [99].

### 4.3. UPLC-Q-TOF-MS Analysis Condition

Chromatographic conditions: Analysis was performed with a Waters CORTECS^®^ UPLC^®^ T3 column (2.1 × 100 mm, 1.6 µm). The mobile phases consist of Solvent A (acetonitrile) and Solvent B (aqueous solution of 0.1% formic acid). The gradient elution protocol is executed as outlined below: 0% A (0–5 min), 0–5% A (5–10 min), 5–15% A (10–20 min), 15–20% A (20–30 min), 20–30% A (30–35 min), 30% A (35–38 min), 30–45% A (38–48 min), 45–90% A (48–55 min), 90% A (55–58 min), 90–0% A (58–58.1 min), 0% A (58.1–60 min). The mobile phase flow rate is 0.3 mL/min, with an injection volume of 2 μL per instance. Wavelength (190–400 nm) and column temperature (30 °C).

Spectrometry conditions: Mass spectrometry analysis was conducted utilizing the AB Sciex TripleTOF^®^ 4600 equipment (SCIEX Company, Framingham, MA, USA). The mass spectrometry detection mode is examined in both positive and negative ion modes, with electrospray ionization (ESI) as the ionization source. The precise conditions are as follows: curtain as pressure at 35 psi; ion source temperature at 500 °C; declustering potential at 100 V; collision energy at 10 eV; collision energy spread at 20 eV; ion release delay at 30 ms; ion release width at 15 ms. The spectrum acquisition ranges of MS1 and MS2 are *m*/*z* 50–1700 and *m*/*z* 50–1250, respectively [100]. Both mass spectra were obtained using full scan and automatic multi-stage fragmentation modes. Data collection utilized Analyst TF 1.7.1 software, whereas data analysis employed Peakview 1.2 software. It is important to compare the mass spectrometry data with the Natural Products HR-MS/MS spectral Library 1.0 database of multi-level mass spectra in different standards and other sources. For compounds excluded in this database, their detection is through literature reports, mass spectroscopy fragmentation techniques, and so on [59].

### 4.4. Ethics and Animals

SPF-grade male Sprague–Dawley rats (210–230 g) were acquired from Shanghai Leigen Laboratory Animal Co., Ltd. (Shanghai, China), under license number SHLG (Hu) 2024-0004. All rats were acclimatized and fed in the SPF experimental animal facility for 7 days before the commencement of the trial. The temperature in the SPF experimental animal room is maintained at 22 ± 1 °C, with a 12 h light-dark cycle. Rats have unrestricted access to food and water [59]. All experiments received ethical approval from the Shanghai Changhai Hospital Ethics Committee (Ethical Approval Number: 20250902; Approval Date: 2 September 2025).

### 4.5. Modeling and Experimental Group Design

Our team has created a specialized experimental system that has received a national utility model patent (No. CN218606938U) for the purpose of producing a rat model of low-temperature injuries in seawater. The complete array of equipment can function constantly in a controlled-temperature laboratory setting and accommodate the processing of numerous experimental animals simultaneously. The fundamental elements of the system comprise a vertically orientated rat fixator independently developed and patented by our research team (No. CN218899782U), a fully immersive seawater tank, and an automated constant temperature control module. A forced circulation design enables seawater to flow swiftly and maintain a constant temperature of 15 °C, thereby eliciting a hypothermic response in awake and confined animals. Following weight-based numbering, the rats underwent randomization for group allocation. The experimental rats were randomly assigned to four groups to ascertain the appropriate dosage of SFD. The control group comprised 30 rats, while the other groups contained 50 rats each. Group 1 was the control group and given distilled water. Group 2 was the SFD (2:1) group, administered SFD (2:1) at doses of 10.8, 5.4, 2.7, 1.35, and 0.675 g/kg, respectively. Group 3 comprised the SFD (1:1) cohort, administered SFD (1:1) at dosages of 10.8, 5.4, 2.7, 1.35, and 0.675 g/kg, respectively. Group 4 received SFD (1:2) at dosages of 10.8, 5.4, 2.7, 1.35, and 0.675 g/kg, respectively. To assess the acute protective effect, after intragastric injection, rats were immediately immersed in simulated seawater at 15 °C.

### 4.6. Survival Analysis

Immersion in simulated seawater can induce adverse reactions in the rats, including shivering and impaired consciousness. We recorded each rat’s electrocardiogram signals at low temperatures. The moment is indicated as soon as an irreversible equipotential straight line appears on the screen, indicating that the animal is dead. We computed each rat’s survival time from the start of this experiment to its death. After summarizing, we used the Kaplan-Meier survival analysis to show how different therapies affected the survival time.

### 4.7. CBT Detection and Analysis

We ran this experiment in a self-paired comparison using 2 groups of 10 rats each: one gavaged with SFD at dosages of 1.35 g/kg, whereas the other was given distilled water. After administering these ingredients, all rats were immersed in simulated seawater. The CBT was measured using a high-accuracy rectal thermometer in normal rats when they were untreated, active, and mentally stable. We used randomized ear tag numbers and holders for the experiment, and the circulating water temperature was fixed at 15 °C. Rats from each group were initially placed on the platform, and immersion times were recorded. CBT was measured each time within 20, 40, 60, 90, 120, 180, 240 min. The CBT of each rat was measured within one minute, and the surrounding temperature was set to 25 °C to minimize the effect of room temperature on hypothermic rats. After sufficient lubrication, the rectal probe was inserted into the rat rectum to about 2 cm. When the reading of the thermometer was stabilized, the probe was withdrawn, and the measurement was recorded. We repeated this procedure for subsequent measurements.

### 4.8. Monitoring of Energy Metabolism

The rat respiratory metabolic measurement system consisted of a data acquisition controller, a cage, a communication module, and filters. The system was used for at least 6 h before the experiment to calibrate the sensors and flow meters. In order to control for circadian effects, monitoring was conducted from 13:00 to 20:00, and rats were fasted for 12 h with a fixed ambient temperature of 25 °C. Rats were placed in the cage to measure resting metabolic rate (RMR) and experimental metabolic rate sequentially. RMR was the average energy metabolic rate over a 90 min period when the rat was silent.

Experimental metabolic rates of rats were measured at 45, 90, 135, and 180 min of hypothermic seawater immersion. Experiments were automatically recorded by the system software and visualized with indicators of the oxygen consumption rate, carbon dioxide production rate, and energy metabolic rates. The metabolic equivalent for both groups was calculated as: (experimental metabolic rates at a measurement)/RMR × 100%, which is a measure of the relative energy metabolic level.

### 4.9. Network Pharmacological Analysis

The plasma-absorbed compounds of SFD were identified from UPLC-Q-TOF-MS. The target genes for hypothermia caused by seawater immersion were mainly from GeneCards. We searched for keywords related to hypothermia due to cold water or seawater in the form “hypothermia exposure in seawater,” “immersed in cold water”, “cold injury soaked in cold waters,” and so on. Once we have retrieved gene targets for all 24 keywords, duplicate entries are removed.

We visualize the overlap of main component targets with potential disease targets in SFD using Venn analysis. In order to further investigate the PPI network of these intersection targets, we downloaded the target list into the STRING database to create the PPI network with a confidence value of 0.9 [59]. The resulting interaction data was then exported and visualized using Cytoscape 3.9.1 software. Within this network, nodes were ranked based on their degree values, where larger and darker nodes indicate higher connectivity and thus greater importance in the network. Additionally, the CytoHubba plugin was applied to analyze the PPI network topologically using the maximal clique centrality algorithm to score and rank the nodes, and we selected the top 10 nodes. Finally, the Metascape database (https://metascape.org/gp/index.html#/main/step1/ (accessed on 3 May 2025)) was used to perform GO analysis and KEGG pathway enrichment analysis for the selected targets [59,101].

### 4.10. Enzyme-Linked Immunosorbent Assay (ELISA)

The concentrations of FFAs and TG in homogenized BAT, along with the levels of NE and cAMP in serum, were quantified utilizing ELISA kits. Absorbance was measured with a microplate reader, and sample concentrations were calculated based on the corresponding standard curves.

### 4.11. RT-PCR Analysis

Total RNA was isolated from BAT utilizing the RNAiso Plus reagent, and its concentration was subsequently measured. Complementary DNA (cDNA) synthesis was performed employing a reverse transcription kit. Quantitative polymerase chain reaction was conducted using SYBR Green chemistry to quantify the mRNA expression levels of UCP1, PPARGC1A (encoding PGC1α), and PPARG (encoding PPARγ). β-actin was employed as the endogenous control. Relative gene expression levels were calculated using the 2^–ΔΔCt^ method. The sequences of the primers used are listed in Table 4.

### 4.12. Western Blot Analysis

Total protein was extracted from BAT utilizing RIPA lysis buffer supplemented with protease inhibitors. Protein concentrations were determined via a BCA protein assay kit. Equivalent quantities of protein samples were resolved by SDS-PAGE and subsequently transferred onto PVDF membranes. The membranes were then blocked with 5% non-fat milk and incubated overnight at 4 °C with specific primary antibodies targeting UCP1, p38 MAPK, PGC1α, PPARγ, HSL, β3-AR, and PKA. Following this, the membranes were incubated with HRP-conjugated secondary antibodies, and protein bands were visualized using an enhanced chemiluminescence detection system. β-actin served as an internal control [59].

### 4.13. H&E Staining

BAT samples were stored in tissue fixative. The samples were dehydrated by graded steps of ethanol in low to high concentrations and immersed in xylene. The tissues were placed in an embedding machine, infiltrated with molten paraffin, and embedded in paraffin blocks. The blocks were sliced into thin slices with a microtome. Sections were mounted on glass slides and dried in an oven. The segments were deparaffinized in xylene and anhydrous ethanol. Sections were stained with hematoxylin for 3–5 min, differentiated, blued, and rinsed. Sections had been washed with 95% ethanol for 1 min and were counterstained with eosin for 15 s. Finally, the section was mounted, tested by a microscope, and images were collected.

### 4.14. Statistical Analysis

The statistical analysis was conducted using SPSS 21.0. For the Kaplan–Meier survival curve analysis, the Log-rank test was used for comparison between groups. Quantitative data were presented as mean ± SD. *T*-tests or Mann–Whitney U tests were employed for two-group comparisons of normally distributed data with equal variances. Significance was defined at *p* < 0.05.

## 5. Conclusions

Seawater-immersion-induced hypothermia poses a potential health threat to maritime personnel, ocean tourists, and naval service members. It occurs frequently during naval combat and maritime disasters and is characterized by unpredictability and catastrophic consequences. Moreover, search and rescue operations at sea are more challenging than those on land, further increasing the risk of mortality among affected individuals. Current research on seawater-immersion-induced hypothermia, both domestically and internationally, has primarily focused on its pathogenesis and rewarming strategies, with few reports on pharmacological interventions. Considering that prophylactic administration, either before falling into the water or immediately after immersion, is critically important for maintaining vital signs, prolonging survival time after immersion, and increasing the likelihood of successful rescue and medical evacuation, we innovatively confirmed that the administration of SFD (1:1, 1.35 g/kg) helps rats to survive in seawater immersion hypothermia and to increase their CBT. The analysis of SFD components entering the bloodstream at this dose, the mass spectrometry analysis of different dose ratios, and the network pharmacology comparison suggest that the effects of SFD for hypothermia were probably multi-component, targeted, and pathway-related. The experiment showed that SFD may contribute to the suppression of seawater-in-water hypothermia in rats by increasing heat production in BAT. This is likely to be achieved through the activation of the upstream signaling pathway NE-(β3-AR)-cAMP-PKA and p38 MAPK/PGC1α/PPARγ, which in turn activates transcription cascades and lipid metabolism-related processes, ultimately promoting the expression of the thermogenic protein UCP1. However, this study has limitations, including the lack of a systematic dose–response curve and toxicological evaluation across a wider range of doses. Moreover, our evidence is primarily correlative, as we did not assess the phosphorylation status of key kinases such as PKA and p38 MAPK. Future research should focus on characterizing the precise therapeutic window of SFD and elucidating the synergistic mechanisms between *Panax ginseng* and *Aconitum carmichaelii*. Identifying the active pharmaceutical ingredients responsible for the anti-hypothermic effect will also be essential for optimizing clinical application.

## Figures and Tables

**Table 1:** Main active constituents in different ratios of Shenfu Decoction (SFD).

NO.	Compound	RT (min)	Exact Mass	Formula	Precursor Type	Peak Area SFD (1:1)	Peak Area SFD (1:2)	Peak Area SFD (2:1)
1	Uridine	2.39	243.0623	C_9_H_12_N_2_O_6_	[M-H]^−^	328,657	418,795	435,402
2	Adenosine	4.61	268.1040	C_10_H_13_N_5_O_4_	[M+H]^+^	159,4976	1,943,824	2,332,433
3	Guanosine	5.35	284.0989	C_10_H_13_N_5_O_5_	[M+H]^+^	226,208	197,467	252,874
4	Karacolidine	10.63	394.2588	C_22_H_35_NO_5_	[M+H]^+^	1,253,105	9,370,746	4,740,174
5	5-[(2-O-β-D-apiofuranosyl-β-D-glucopyranosyl)oxy]-2-hydroxybenzoic acid	12.51	447.1144	C_18_H_24_O_13_	[M-H]^−^	465,215	318,560	489,984
6	Mesaconine	13.96	486.2698	C_24_H_39_NO_9_	[M+H]^+^	1,490,325	18,594,727	10,784,333
7	Carmichaeline	14.47	378.2639	C_22_H_35_NO_4_	[M+H]^+^	296,846	2,252,136	1,023,286
8	Talatizidine	14.67	408.2745	C_23_H_37_NO_5_	[M+H]^+^	185,009	646,752	274,813
9	Songorine	15.06	358.2377	C_22_H_31_NO_3_	[M+H]^+^	1,467,666	8,719,785	4,586,230
10	Eucomic acid	15.14	239.0561	C_11_H_12_O_6_	[M-H]^−^	1,346,017	825,896	338,505
11	Aconine	15.31	500.2854	C_25_H_41_NO_9_	[M+H]^+^	256,955	2,328,107	1,234,437
12	Fuziline	16.44	454.2799	C_24_H_39_NO_7_	[M+H]^+^	4,975,107	49,469,728	24,417,458
13	Neoline	17.23	438.2850	C_24_H_39_NO_6_	[M+H]^+^	1,791,264	8,078,738	3,911,224
14	(+)-N-methyllaurotetanine	18.12	342.1700	C_20_H_23_NO_4_	[M+H]^+^	1,556,140	5,295,679	3,010,992
15	Talatisamine	18.93	422.2901	C_24_H_39_NO_5_	[M+H]^+^	554,344	5,475,634	2,559,854
16	Chasmanine	20.48	452.3007	C_25_H_41_NO_6_	[M+H]^+^	305,480	4,137,947	1,811,039
17	(3β,6β,12β)-3,12,25-Trihydroxydammarane-6,20-diyl bis[β-D-glucopyranoside]	21.31	863.5010	C_42_H_74_O_15_	[M+FA-H]^−^	57,798	47,449	73,254
18	14-Benzoyl-10-hydroxymesaconine	22.31	606.2909	C_31_H_43_NO_11_	[M+H]^+^	1,214,251	8,336,534	4,071,076
19	14-Benzoyl-10-hydroxyaconine	24.39	620.3065	C_32_H_45_NO_11_	[M+H]^+^	411,360	3,066,916	1,351,484
20	Benzoylmesaconine	26.26	590.2960	C_31_H_43_NO_10_	[M+H]^+^	19,429,226	158,437,047	79,522,984
21	Benzoylaconine	28.85	604.3116	C_32_H_45_NO_10_	[M+H]^+^	3,929,009	21,038,648	9,901,974
22	Benzoylhypaconine	30.68	574.3011	C_31_H_43_NO_9_	[M+H]^+^	4,636,457	29,012,873	13,619,581
23	Ginsenoside Rg1	31.12	845.4904	C_42_H_72_O_14_	[M+FA-H]^−^	2,158,838	2,254,128	3,339,707
24	Ginsenoside Re	31.52	991.5483	C_48_H_82_O_18_	[M+FA-H]^−^	630,516	732,182	1,259,283
25	Pyromesaconitine	32.34	572.2854	C_31_H_41_NO_9_	[M+H]^+^	223,037	12,229,137	5,459,006
26	14-Benzoyldeoxyaconine	33.08	588.3167	C_32_H_45_NO_9_	[M+H]^+^	872,211	5,133,299	2,315,045
27	16-Epipyrohypaconitine	34.96	556.2905	C_31_H_41_NO_8_	[M+H]^+^	99,566	5,345,443	2,256,744
28	Ginsenoside Rf	36.07	845.4904	C_42_H_72_O_14_	[M+FA-H]^−^	1,362,760	1,198,195	1,851,290
29	14-O-Anisoylneoline	36.49	572.3218	C_32_H_45_NO_8_	[M+H]^+^	638,562	1,588,373	698,072
30	Hypaconitine	36.68	616.3116	C_33_H_45_NO_10_	[M+H]^+^	3,120,014	213,954	50,731
31	Ginsenoside F5	36.68	815.4798	C_41_H_70_O_13_	[M+FA-H]^−^	661,789	511,368	716,223
32	Ginsenoside F1	37.42	683.4376	C_36_H_62_O_9_	[M+FA-H]^−^	2,055,289	1,629,964	2,454,681
33	Ginsenoside Rg2	37.70	829.4955	C_42_H_72_O_13_	[M+FA-H]^−^	1,267,474	1,018,499	1,944,620
34	Ginsenoside Rh1	37.93	683.4376	C_36_H_62_O_9_	[M+FA-H]^−^	1,403,243	946,264	1,450,613
35	Ginsenoside Rb1	38.01	1153.6011	C_54_H_92_O_23_	[M+FA-H]^−^	494,991	392,384	669,386
36	Ginsenoside Ro	38.68	955.4908	C_48_H_76_O_19_	[M-H]^−^	1,477,818	659,330	1,203,468
37	Ginsenoside Rc	38.84	1123.5906	C_53_H_90_O_22_	[M+FA-H]^−^	206,154	178,908	267,545
38	Ginsenoside Rb2	39.87	1123.5906	C_53_H_90_O_22_	[M+FA-H]^−^	356,928	232,981	447,415
39	Quinquenoside R1	41.16	1195.6117	C_56_H_94_O_24_	[M+FA-H]^−^	50,962	37,924	76,712
40	Chikusetsusaponin-Iva	41.42	793.4380	C_42_H_66_O_14_	[M-H]^−^	95,619	48,826	139,141
41	Ginsenoside Rd	41.73	991.5483	C_48_H_82_O_18_	[M+FA-H]^−^	364,454	266,785	606,196
42	Ginsenoside Rg6	44.53	811.4849	C_42_H_70_O_12_	[M+FA-H]^−^	153,698	136,218	325,871
43	Ginsenoside F4	45.00	811.4849	C_42_H_70_O_12_	[M+FA-H]^−^	226,391	168,334	451,565
44	Ginsenoside Rk3	45.20	665.4270	C_36_H_60_O_8_	[M+FA-H]^−^	529,510	403,453	817,759
45	Ginsenoside Rh4	45.76	665.4270	C_36_H_60_O_8_	[M+FA-H]^−^	787,737	542,166	1,215,263
46	Zingibroside R1	46.56	793.4380	C_42_H_66_O_14_	[M+FA-H]^−^	140,237	55,610	224,269
47	20(S)-Ginsenoside Rg3	47.85	829.4955	C_42_H_72_O_13_	[M+FA-H]^−^	642,196	331,612	1,124,033
48	20(R)-Ginsenoside Rg3	48.21	829.4955	C_42_H_72_O_13_	[M+FA-H]^−^	1,390,339	593,533	1,859,111
49	Ginsenoside Rs3	50.51	871.5061	C_44_H_74_O_14_	[M+FA-H]^−^	24,022	12,853	54,689
50	20(R)-Ginsenoside Rs3	50.77	871.5061	C_44_H_74_O_14_	[M+FA-H]^−^	79,541	31,751	110,685
51	Ginsenoside Rg5	51.33	811.4849	C_42_H_70_O_12_	[M+FA-H]^−^	223,020	96,494	429,346
52	Ginsenoside Rk1	51.57	811.4849	C_42_H_70_O_12_	[M+FA-H]^−^	918,208	408,679	1,329,256
53	Ginsenoside Rs5	52.65	853.4955	C_44_H_72_O_13_	[M+FA-H]^−^	55,314	14,197	96,131
54	Ginsenoside Rs4	52.89	853.4955	C_44_H_72_O_13_	[M+FA-H]^−^	26,169	10,180	30,384

**Table 2:** Main plasma-absorbed prototype compounds in Shenfu Decoction (SFD).

NO.	Compound	RT (min)	Exact Mass	Formula	Precursor Type	Peak Area SFD
P1	Karacolidine Isomer	9.07	394.2580	C_22_H_35_NO_5_	[M+H]^+^	79,164
P2	Karacolidine	11.32	394.2582	C_22_H_35_NO_5_	[M+H]^+^	400,879
P3	Mesaconine	14.81	486.2691	C_24_H_39_NO_9_	[M+H]^+^	4,674,467
P4	Carmichaeline	15.34	378.2614	C_22_H_35_NO_4_	[M+H]^+^	35,938
P5	Eucomic acid	15.75	239.0573	C_11_H_12_O_6_	[M-H]^−^	25,459
P6	Songorine	16.24	358.2373	C_22_H_31_NO_3_	[M+H]^+^	394,898
P7	Aconine	16.32	500.2858	C_25_H_41_NO_9_	[M+H]^+^	535,442
P8	Talatizidine	16.60	408.2729	C_23_H_37_NO_5_	[M+H]^+^	54,883
P9	Fuziline	17.16	454.2790	C_24_H_39_NO_7_	[M+H]^+^	2,072,599
P10	Talatizidine Isomer	18.23	408.2729	C_23_H_37_NO_5_	[M+H]^+^	63,115
P11	Neoline	18.61	438.2849	C_24_H_39_NO_6_	[M+H]^+^	130,265
P12	Fuziline Isomer	19.47	454.2789	C_24_H_39_NO_7_	[M+H]^+^	308,314
P13	Talatisamine	20.50	422.2901	C_24_H_39_NO_5_	[M+H]^+^	521,132
P14	14-Benzoyl-10-hydroxymesaconine	24.35	606.2907	C_31_H_43_NO_11_	[M+H]^+^	121,833
P15	14-Benzoyl-10-hydroxyaconine	26.89	620.3081	C_32_H_45_NO_11_	[M+H]^+^	39,838
P16	Benzoylmesaconine	29.79	590.2968	C_31_H_43_NO_10_	[M+H]^+^	4,228,164
P17	Benzoylaconine	32.25	604.3125	C_32_H_45_NO_10_	[M+H]^+^	327,112
P18	Benzoylhypaconine	33.15	574.3027	C_31_H_43_NO_9_	[M+H]^+^	376,940
P19	Ginsenoside Rg1	33.72	845.4953	C_42_H_72_O_14_	[M+FA-H]^−^	3585
P20	Ginsenoside Re	33.88	991.5540	C_48_H_82_O_18_	[M+FA-H]^−^	1096
P21	14-Benzoyldeoxyaconine	35.36	588.3180	C_32_H_45_NO_9_	[M+H]^+^	57,444
P22	Ginsenoside F1	39.56	683.4376	C_36_H_62_O_9_	[M+H]^+^	1169
P23	Ginsenoside Rh1	40.37	683.4376	C_36_H_62_O_9_	[M+FA-H]^−^	1028
P24	Ginsenoside Rb1	41.20	1153.6005	C_54_H_92_O_23_	[M+FA-H]^−^	17,423
P25	Ginsenoside Ro	41.93	955.4903	C_48_H_76_O_19_	[M+FA-H]^−^	1780
P26	Ginsenoside Rb2	42.76	1123.5900	C_53_H_90_O_22_	[M-H]^−^	5990
P27	Ginsenoside Rd	44.22	991.5483	C_48_H_82_O_18_	[M+FA-H]^−^	7833
P28	20(S)-Ginsenoside Rg3	50.20	829.4955	C_42_H_72_O_13_	[M+FA-H]^−^	4175

**Table 3:** Main plasma-absorbed metabolic compounds in Shenfu Decoction (SFD).

NO.	Compound	RT (min)	Precursor Type	Peak Area SFD
M1	Neoline + Demethylation	12.64	[M+H]^+^	103,351
M2	Talatizidine + Demethylation	13.04	[M+H]^+^	42,420
M3	Neoline + Demethylation	14.31	[M+H]^+^	18,728
M4	Neoline + Demethylation	15.29	[M+H]^+^	3482
M5	Neoline + Demethylation	17.60	[M-H]^−^	61,772
M6	Karacolidine + dehydroxylation	17.83	[M+H]^+^	36,449

**Table 4:** Primers sequence information.

Gene	Forward	Reverse
UCP1	TCCCCTGCCATTTACTGTCA	ATCTCGTTTTTACCACATCCACC
PPARG	CCTCTCTGTGATGGATGACCACT	GCTCTTGTGAACGGGATGTCTT
PPARGC1A	CAACTCAGCAAGTCCTCAGTG	ATCACCAAACAGCCGTAGACT
β-actin	CAGCAAGCAGGAGTACGATGAG	TCAAAGAAAGGGTGTAAAACGCA

## Data Availability

The original contributions presented in this study are included in the article. Further inquiries can be directed to the corresponding authors.

## Abbreviations

The following abbreviations are used in this manuscript:

CBT	Core body temperature
TCM	Traditional Chinese Medicine
SFD	Shenfu Decoction
UPLC-Q-TOF-MS	Ultra-Performance Liquid Chromatography-Quadrupole Time-of-Flight-Mass Spectrometry
BPC	Base peak chromatogram
PPI	Protein–protein Interaction
GO	Gene Ontology
KEGG	Kyoto Encyclopedia of Genes and Genomes
FFAs	Free fatty acids
TG	Triglycerides
cDNA	Complementary DNA
UCP1	Uncoupling Protein 1
PPARγ	Peroxisome Proliferator-Activated Receptor Gamma
PGC1α	Peroxisome Proliferator-Activated Receptor Gamma Coactivator 1-Alpha
HSL	Hormone-Sensitive Lipase
NE	Norepinephrine
β3-AR	Beta-3 Adrenergic Receptor
cAMP	Cyclic Adenosine Monophosphate
PKA	Protein Kinase A
ELISA	Enzyme-Linked Immunosorbent Assay
RT-PCR	Reverse Transcription-Polymerase Chain Reaction
WB	Western Blot
MAPK	Mitogen-activated protein kinase
BAT	Brown adipose tissue

## References

[1] Okumura H., Okada N., Hamanaka K., Okada Y., Kitamura T., Matsuyama T. (2025). Electrocardiographic patterns of accidental hypothermia. Am. J. Emerg. Med..

[2] Mydske S., Brattebø G., Helland A.M., Wiggen Ø., Aßmus J., Thomassen Ø. (2025). Treatment of accidental hypothermia: Impact of insulation placement above or below an active external rewarming device on temperature and burn risk. J. Therm. Biol..

[3] Balvers K., Van der Horst M., Graumans M., Boer C., Binnekade J.M., Goslings J.C., Juffermans N.P. (2016). Hypothermia as a predictor for mortality in trauma patients at admittance to the Intensive Care Unit. J. Emerg. Trauma Shock..

[4] Chen R., Yin P., Wang L., Liu C., Niu Y., Wang W., Jiang Y., Liu Y., Liu J., Qi J. (2018). Association between ambient temperature and mortality risk and burden: Time series study in 272 main Chinese cities. BMJ.

[5] Xie Y., Zhou Z., Sun Q., Zhao M., Pu J., Li Q., Sun Y., Dai H., Li T. (2024). Social-economic transitions and vulnerability to extreme temperature events from 1960 to 2020 in Chinese cities. iScience.

[6] Gasparrini A., Guo Y., Hashizume M., Lavigne E., Zanobetti A., Schwartz J., Tobias A., Tong S., Rocklöv J., Forsberg B. (2015). Mortality risk attributable to high and low ambient temperature: A multicountry observational study. Lancet.

[7] Wang L.L., Tian Y.M., Hu S., Zhang H.P., Meng X.X., Zhang H.P., Zhong Y.X., Du M.H., Ding Y. (2023). Study on an Animal Model of Seawater Immersion Injury Following Hemorrhagic Shock. J. Surg. Res..

[8] Iida A., Nakashima T., Okada S. (2023). Development of an estimation method for accidental hypothermia risk due to cold exposure in disasters triggered by earthquakes and tsunamis. Int. J. Disaster Risk Reduct..

[9] Li D., Ma W., Xiong M., Xie P., Feng Y., Liu D., Qiao Y., Shi C. (2023). Water Rewarming After Seawater Hypothermia Mitigates IL-1β in Both Intestinal Tissue and Blood. Ther. Hypothermia Temp. Manag..

[10] Werner L.M., Kevorkian R.T., Getnet D., Rios K.E., Hull D.M., Robben P.M., Cybulski R.J., Bobrov A.G. (2025). Hypothermia: Pathophysiology and the propensity for infection. Am. J. Emerg. Med..

[11] Liu K.L., Yu X.J., Sun T.Z., Wang Y.C., Chen M.X., Su Y.W., Zhang H.C., Chen Y.M., Gao H.L., Shi X.L. (2020). Effects of seawater immersion on open traumatic brain injury in rabbit model. Brain Res..

[12] Musi M.E., Sheets A., Zafren K., Brugger H., Paal P., Hölzl N., Pasquier M. (2021). Clinical staging of accidental hypothermia: The Revised Swiss System: Recommendation of the International Commission for Mountain Emergency Medicine (ICAR MedCom). Resuscitation.

[13] Bitzer K., Breindahl N., Kelly B., Sørensen O.B., Laugesen M., Wolthers S.A., Blomberg S.N.F., Steinmetz J., Wiberg S., Christensen H.C. (2025). The role of accidental hypothermia in drowning patients with out-of-hospital cardiac arrest: A nationwide registry-based cohort study. Resuscitation.

[14] Junaid M., Mahmud-Or-Rashid M. (2024). Computational insights into survival durations and prehospital interventions in accidental cold-water immersion: A comprehensive analysis of fresh and saltwater temperatures. Heliyon.

[15] Polderman K.H. (2009). Mechanisms of action, physiological effects, and complications of hypothermia. Crit. Care Med..

[16] Podsiadło P., Smoleń A., Brožek T., Kosiński S., Balik M., Hymczak H., Cools E., Walpoth B., Nowak E., Dąbrowski W. (2023). Extracorporeal Rewarming Is Associated with Increased Survival Rate in Severely Hypothermic Patients with Preserved Spontaneous Circulation. ASAIO J..

[17] Bennett B.L., Holcomb J.B. (2017). Battlefield Trauma-Induced Hypothermia: Transitioning the Preferred Method of Casualty Rewarming. Wilderness Environ. Med..

[18] Yoneshiro T., Matsushita M., Sakai J., Saito M. (2025). Brown fat thermogenesis and cold adaptation in humans. J. Physiol. Anthropol..

[19] Keipert S., Gaudry M.J., Kutschke M., Keuper M., Dela Rosa M.A.S., Cheng Y., Monroy Kuhn J.M., Laterveer R., Cotrim C.A., Giere P. (2024). Two-stage evolution of mammalian adipose tissue thermogenesis. Science.

[20] Okamatsu-Ogura Y., Kuroda M., Tsutsumi R., Tsubota A., Saito M., Kimura K., Sakaue H. (2020). UCP1-dependent and UCP1-independent metabolic changes induced by acute cold exposure in brown adipose tissue of mice. Metabolism.

[21] Yeo H., Lim J.H., Eom J., Kim M., Kwon H., Kang S.W., Song Y. (2024). Diet-induced obesity and aging-induced upregulation of Trib3 interfere with energy homeostasis by downregulating the thermogenic capacity of BAT. Exp. Mol. Med..

[22] Johnson J.M., Peterlin A.D., Balderas E., Sustarsic E.G., Maschek J.A., Lang M.J., Jara-Ramos A., Panic V., Morgan J.T., Villanueva C.J. (2023). Mitochondrial phosphatidylethanolamine modulates UCP1 to promote brown adipose thermogenesis. Sci. Adv..

[23] Chang S.H., Jang J., Oh S., Yoon J.H., Jo D.G., Yun U.J., Park K.W. (2021). Nrf2 induces Ucp1 expression in adipocytes in response to β3-AR stimulation and enhances oxygen consumption in high-fat diet-fed obese mice. BMB Rep..

[24] Cero C., Lea H.J., Zhu K.Y., Shamsi F., Tseng Y.H., Cypess A.M. (2021). β3-Adrenergic receptors regulate human brown/beige adipocyte lipolysis and thermogenesis. JCI Insight.

[25] Diané A., Nikolic N., Rudecki A.P., King S.M., Bowie D.J., Gray S.L. (2014). PACAP is essential for the adaptive thermogenic response of brown adipose tissue to cold exposure. J. Endocrinol..

[26] Nie Y., Yan Z., Yan W., Xia Q., Zhang Y. (2015). Cold exposure stimulates lipid metabolism, induces inflammatory response in the adipose tissue of mice and promotes the osteogenic differentiation of BMMSCs via the p38 MAPK pathway *in vitro*. Int. J. Clin. Exp. Pathol..

[27] Bordicchia M., Liu D., Amri E.Z., Ailhaud G., Dessì-Fulgheri P., Zhang C., Takahashi N., Sarzani R., Collins S. (2012). Cardiac natriuretic peptides act via p38 MAPK to induce the brown fat thermogenic program in mouse and human adipocytes. J. Clin. Investig..

[28] Wang T., Sun X., Zhang Y., Wang Q., Cheng W., Gao Y., Shi X., Jin J. (2025). Baicalin Promotes Skeletal Muscle Fiber Remodeling by Activating the p38MAPK/PGC-1α Signaling Pathway. J. Agric. Food Chem..

[29] Dumesic P.A., Wilensky S.E., Bose S., Van Vranken J.G., Gygi S.P., Spiegelman B.M. (2025). RBM43 controls PGC1α translation and a PGC1α-STING signaling axis. Cell Metab..

[30] Song P., Zhao J., Li F., Zhao X., Feng J., Su Y., Wang B., Zhao J. (2024). Vitamin A regulates mitochondrial biogenesis and function through p38 MAPK-PGC-1α signaling pathway and alters the muscle fiber composition of sheep. J. Anim. Sci. Biotechnol..

[31] Sahún-Español Á., Clemente C., Jiménez-Loygorri J.I., Sierra-Filardi E., Herrera-Melle L., Gómez-Durán A., Sabio G., Monsalve M., Boya P., Arroyo A.G. (2022). p38 MAPK priming boosts VSMC proliferation and arteriogenesis by promoting PGC1α-dependent mitochondrial dynamics. Sci. Rep..

[32] Choe H.J., Lee J.S., Park J.Y., Lee S.A., Park Y.J., Chung S.S., Park K.S. (2025). SENP2 regulates UCP1-dependent thermogenesis in brown adipocytes via deSUMOylation of ERRα. Exp. Mol. Med..

[33] Li S.J., Wei J.Q., Kang Y.Y., Wang R.Q., Rong W.W., Zhao J.J., Deng Q.W., Gao P.J., Li X.D., Wang J.G. (2024). Natriuretic peptide receptor-C perturbs mitochondrial respiration in white adipose tissue. J. Lipid Res..

[34] Cremonini E., Da Silva L.M.E., Lanzi C.R., Marino M., Iglesias D.E., Oteiza P.I. (2024). Anthocyanins and their metabolites promote white adipose tissue beiging by regulating mitochondria thermogenesis and dynamics. Biochem. Pharmacol..

[35] Shen H., He T., Wang S., Hou L., Wei Y., Liu Y., Mo C., Zhao Z., You W., Guo H. (2022). SOX4 promotes beige adipocyte-mediated adaptive thermogenesis by facilitating PRDM16-PPARγ complex. Theranostics.

[36] Boström P., Wu J., Jedrychowski M.P., Korde A., Ye L., Lo J.C., Rasbach K.A., Boström E.A., Choi J.H., Long J.Z. (2012). A PGC1-α-dependent myokine that drives brown-fat-like development of white fat and thermogenesis. Nature.

[37] Ma W., Wang M., Chen J., Wang Y., Chen J., Pei Y., Gong Y., You J., Cao Y., Zhou J. (2025). Qingshu Yiqi decoction ameliorates exertional heat stroke-induced intestinal barrier injury via NF-κB/MLC pathway and gut microbiota. Phytomedicine.

[38] Zhang H., Jin B., You X., Yi P., Guo H., Niu L., Yin Q., Shi J., Zhang Y., Zhuang P. (2023). Pharmacodynamic advantages and characteristics of traditional Chinese medicine in prevention and treatment of ischemic stroke. Chin. Herb. Med..

[39] Zhou T., Zhang C., Wang X., Lin J., Yu J., Liang Y., Guo H., Yang M., Shen X., Li J. (2024). Research on traditional Chinese medicine as an effective drug for promoting wound healing. J. Ethnopharmacol..

[40] Zhang W., Ren C., Yang Y., Xu J., Tong F., Wu X., Yang Y. (2024). Ginseng aconitum decoction (Shenfu Tang) provides neuroprotection by ameliorating impairment of blood-brain barrier in cerebral ischemia-reperfusion injury. Brain Res..

[41] Xu Y.W., Xu Z.D., An R., Zhang H., Wang X.H. (2020). Revealing the synergistic mechanism of Shenfu Decoction for anti-heart failure through network pharmacology strategy. Chin. J. Nat. Med..

[42] Luo J., Min S., Wei K., Cao J. (2008). Ion channel mechanism and ingredient bases of Shenfu Decoction’s cardiac electrophysiological effects. J. Ethnopharmacol..

[43] Yang B., Wang S., Yang Y., Wang Y. (2025). Toxicity and safety profile evaluation of Shenfu injection in a murine sepsis model. J. Ethnopharmacol..

[44] Huang P., Guo Y., Hu X., Fang X., Xu X., Liu Q. (2024). Mechanism of Shenfu injection in suppressing inflammation and preventing sepsis-induced apoptosis in murine cardiomyocytes based on network pharmacology and experimental validation. J. Ethnopharmacol..

[45] Li X., Lin H., Wang Q., Cui L., Luo H., Luo L. (2020). Chemical composition and pharmacological mechanism of shenfu decoction in the treatment of novel coronavirus pneumonia (COVID-19). Drug Dev. Ind. Pharm..

[46] Wang Y.J., Chen H.Z., Wang Z.B., Sun C.Y., Guo C.Y., Ruan Y., Li C.T., Zou B., Yin Z.F., Gu W. (2025). Shenfu Decoction Extends Survival Time of Seawater-Induced Hypothermia in Rats: The Role of Metabolomics and Gut Microbiota. Curr. Drug Metab..

[47] Tian D., Yang Y., Yu M., Han Z.Z., Wei M., Zhang H.W., Jia H.M., Zou Z.M. (2020). Anti-inflammatory chemical constituents of Flos Chrysanthemi Indici determined by UPLC-MS/MS integrated with network pharmacology. Food Funct..

[48] Jiao G., Wang Y., Song Y., Chen Y., Fan X., Zhao Q., Pang T., Zhang F., Chen W. (2024). Combination of ultra-performance liquid chromatography-quadrupole time-of-flight mass spectrometry and network pharmacology to reveal the key effective compounds and mechanism of Shengxian decoction for ameliorating doxorubicin cardiotoxicity. J. Sep. Sci..

[49] Chen Y., Yu R., Jiang L., Zhang Q., Li B., Liu H., Xu G. (2019). A Comprehensive and Rapid Quality Evaluation Method of Traditional Chinese Medicine Decoction by Integrating UPLC-QTOF-MS and UFLC-QQQ-MS and its Application. Molecules.

[50] Gao Z., Wang J., Lu G., Wu Q., Wang S., Wu X., Ou C., Wu Z., Yu H., Wang Y. (2024). Exploration the mechanism of Shenling Baizhu San in the treatment of chronic obstructive pulmonary disease based on UPLC-Q-TOF-MS/MS, network pharmacology and *in vitro* experimental verification. J. Ethnopharmacol..

[51] Ablajan N., Zhao B., Wenjuan X., Zhao J., Sagdullaev S.S., Guoan Z., Aisa H.A. (2023). Chemical components of *Aconitum barbatum* var. *puberulum* and their cytotoxic and antibacterial activities. Nat. Prod. Res..

[52] Gao W., Liu X.G., Liu L., Li P., Yang H. (2018). Targeted profiling and relative quantification of benzoyl diterpene alkaloids in *Aconitum* roots by using LC-MS/MS with precursor ion scan. J. Sep. Sci..

[53] Sun Q., Cao H., Zhou Y., Wang X., Jiang H., Gong L., Yang Y., Rong R. (2016). Qualitative and quantitative analysis of the chemical constituents in Mahuang-Fuzi-Xixin decoction based on high performance liquid chromatography combined with time-of-flight mass spectrometry and triple quadrupole mass spectrometers. Biomed. Chromatogr..

[54] Jiang Z.B., Jiang B.Y., Zhu C.G., Guo Q.L., Peng Y., Wang X.L., Lin S., Shi J.G. (2014). Aromatic acid derivatives from the lateral roots of *Aconitum carmichaelii*. J. Asian Nat. Prod. Res..

[55] Sun B.S., Gu L.J., Fang Z.M., Wang C.Y., Wang Z., Lee M.R., Li Z., Li J.J., Sung C.K. (2009). Simultaneous quantification of 19 ginsenosides in black ginseng developed from *Panax ginseng* by HPLC-ELSD. J. Pharm. Biomed. Anal..

[56] Liu Y., Li J., He J., Abliz Z., Qu J., Yu S., Ma S., Liu J., Du D. (2009). Identification of new trace triterpenoid saponins from the roots of *Panax notoginseng* by high-performance liquid chromatography coupled with electrospray ionization tandem mass spectrometry. Rapid Commun. Mass Spectrom..

[57] Yang Y., Yin X.J., Guo H.M., Wang R.L., Song R., Tian Y., Zhang Z.J. (2014). Identification and comparative analysis of the major chemical constituents in the extracts of single fuzi herb and fuzi-gancao herb-pair by UFLC-IT-TOF/MS. Chin. J. Nat. Med..

[58] Zhang X., Cui J., Sun J., Fan B., Wang F., Lu C. (2025). Protective Effects of Arecoline on LPS-Induced Neuroinflammation in BV2 Microglial Cells. Int. J. Mol. Sci..

[59] Chen W., Xu L., Wang L., Shan Y.N., Li Y., Zhu J.S. (2025). Qing-Re-Hua-Shi Decoction ameliorates DSS-induced colitis by modulating multiple signaling pathways and remodeling the gut microbiota and metabolite profile. Front. Cell. Infect. Microbiol..

[60] Sun Y., Xia Y., Ge W., Li Q. (2026). Tirzepatide reduces intracellular lipid content by promoting the browning of white fat via the cAMP signaling pathway. Eur. J. Pharmacol..

[61] Wang L., Lei Z., Zhang G., Cheng Y., Zhong M., Zhang G., Hu S. (2024). Olodaterol promotes thermogenesis in brown adipocytes via regulation of the β2-AR/cAMP/PKA signaling pathway. Biochem. Biophys. Res. Commun..

[62] Lian H., Zhou L., Zhang Y., Song Y.H., Zhang Y.M., Cao Z.H., Wang Z.Y. (2021). Increased energy expenditure and activated β3-AR-cAMP-PKA signaling pathway in the interscapular brown adipose tissue of 6-OHDA-induced Parkinson’s disease model rats. Anat. Rec..

[63] Young A.J., Muza S.R., Sawka M.N., Gonzalez R.R., Pandolf K.B. (1986). Human thermoregulatory responses to cold air are altered by repeated cold water immersion. J. Appl. Physiol..

[64] Zhang Z., Yang D., Xiang J., Zhou J., Cao H., Che Q., Bai Y., Guo J., Su Z. (2021). Non-Shivering Thermogenesis Signalling Regulation and Potential Therapeutic Applications of Brown Adipose Tissue. Int. J. Biol. Sci..

[65] Argentato P.P., de Cássia César H., Estadella D., Pisani L.P. (2018). Programming mediated by fatty acids affects uncoupling protein 1 (UCP-1) in brown adipose tissue. Br. J. Nutr..

[66] Stach P., Skowron K., Rojek S., Cios A., Wesołowska A., Huestis M.A., Gil K. (2026). Impact of environmentally induced hypothermia on fentanyl and norfentanyl pharmacokinetics following intravenous administration to Wistar rats. Pharmacol. Rep..

[67] van der Veer M.A.A., de Haan T.R., Franken L.G.W., van Hest R.M., Groenendaal F., Dijk P.H., de Boode W.P., Simons S., Dijkman K.P., van Straaten H.L.M. (2024). Population pharmacokinetics of vancomycin in term neonates with perinatal asphyxia treated with therapeutic hypothermia. Br. J. Clin. Pharmacol..

[68] Morrison S.F., Nakamura K. (2019). Central Mechanisms for Thermoregulation. Annu. Rev. Physiol..

[69] Wira C.R., Becker J.U., Martin G., Donnino M.W. (2008). Anti-arrhythmic and vasopressor medications for the treatment of ventricular fibrillation in severe hypothermia: A systematic review of the literature. Resuscitation.

[70] Danzl D. (2002). Hypothermia. Semin. Respir. Crit. Care Med..

[71] Hong B.N., Do M.H., Her Y.R., Lee Y.R., Kang T.H. (2015). The Effects of *Panax ginseng* and *Panax quinquefolius* on Thermoregulation in Animal Models. Evid.-Based Complement. Altern. Med..

[72] Zhang X.Y., Khakisahneh S., Han S.Y., Song E.J., Nam Y.D., Kim H. (2024). Ginseng extracts improve circadian clock gene expression and reduce inflammation directly and indirectly through gut microbiota and PI3K signaling pathway. npj Biofilms Microbiomes.

[73] Lee Y.Y., Saba E., Irfan M., Kim M., Chan J.Y., Jeon B.S., Choi S.K., Rhee M.H. (2019). The anti-inflammatory and anti-nociceptive effects of Korean black ginseng. Phytomedicine.

[74] Makino T., Kato K., Mizukami H. (2009). Processed aconite root prevents cold-stress-induced hypothermia and immuno-suppression in mice. Biol. Pharm. Bull..

[75] Liu J., Tan Y., Ao H., Feng W., Peng C. (2021). Aqueous extracts of Aconite promote thermogenesis in rats with hypothermia via regulating gut microbiota and bile acid metabolism. Chin. Med..

[76] Zhang D., Cheng H., Wu J., Zhou Y., Tang F., Liu J., Feng W., Peng C. (2024). The energy metabolism-promoting effect of aconite is associated with gut microbiota and bile acid receptor TGR5-UCP1 signaling. Front. Pharmacol..

[77] Cao W., Medvedev A.V., Daniel K.W., Collins S. (2001). beta-Adrenergic activation of p38 MAP kinase in adipocytes: cAMP induction of the uncoupling protein 1 (UCP1) gene requires p38 MAP kinase. J. Biol. Chem..

[78] Xu Y., Wang N., Tan H.Y., Li S., Zhang C., Zhang Z., Feng Y. (2020). *Panax notoginseng* saponins modulate the gut microbiota to promote thermogenesis and beige adipocyte reconstruction via leptin-mediated AMPKα/STAT3 signaling in diet-induced obesity. Theranostics.

[79] Pellegrino M.J., McCully B.H., Habecker B.A. (2014). Leptin stimulates sympathetic axon outgrowth. Neurosci. Lett..

[80] Rahmouni K., Sigmund C.D., Haynes W.G., Mark A.L. (2009). Hypothalamic ERK mediates the anorectic and thermogenic sympathetic effects of leptin. Diabetes.

[81] Packer M. (2023). Qiliqiangxin: A multifaceted holistic treatment for heart failure or a pharmacological probe for the identification of cardioprotective mechanisms?. Eur. J. Heart Fail..

[82] Huang Q., Lou T., Lu J., Wang M., Chen X., Xue L., Tang X., Qi W., Zhang Z., Su H. (2022). Major ginsenosides from *Panax ginseng* promote aerobic cellular respiration and SIRT1-mediated mitochondrial biosynthesis in cardiomyocytes and neurons. J. Ginseng Res..

[83] Li J.B., Zhang R., Han X., Piao C.L. (2018). Ginsenoside Rg1 inhibits dietary-induced obesity and improves obesity-related glucose metabolic disorders. Braz. J. Med. Biol. Res..

[84] Li Y., Guan Y., Wang Y., Yu C.L., Zhai F.G., Guan L.X. (2017). Neuroprotective Effect of the Ginsenoside Rg1 on Cerebral Ischemic Injury *In Vivo* and *In Vitro* Is Mediated by PPARγ-Regulated Antioxidative and Anti-Inflammatory Pathways. Evid.-Based Complement. Altern. Med..

[85] Zhang Z., Yang K., Mao R., Zhong D., Xu Z., Xu J., Xiong M. (2022). Ginsenoside Rg1 inhibits oxidative stress and inflammation in rats with spinal cord injury via Nrf2/HO-1 signaling pathway. Neuroreport.

[86] Luo M., Yan D., Sun Q., Tao J., Xu L., Sun H., Zhao H. (2020). Ginsenoside Rg1 attenuates cardiomyocyte apoptosis and inflammation via the TLR4/NF-kB/NLRP3 pathway. J. Cell. Biochem..

[87] Gao H., Li Z., Cheng C., Cui J., Peng J., Wang X., Zhang M., Hou Y., Bai G. (2023). Fuziline Ameliorates Glucose and Lipid Metabolism by Activating Beta Adrenergic Receptors to Stimulate Thermogenesis. Int. J. Mol. Sci..

[88] Cho H.T., Kim J.H., Lee J.H., Kim Y.J. (2017). Effects of *Panax ginseng* extracts prepared at different steaming times on thermogenesis in rats. J. Ginseng Res..

[89] Su D., Jiang T., Song Y., Li D., Zhan S., Zhong T., Guo J., Li L., Zhang H., Wang L. (2025). Identification of a distal enhancer of Ucp1 essential for thermogenesis and mitochondrial function in brown fat. Commun. Biol..

[90] Wang G., Meyer J.G., Cai W., Softic S., Li M.E., Verdin E., Newgard C., Schilling B., Kahn C.R. (2019). Regulation of UCP1 and Mitochondrial Metabolism in Brown Adipose Tissue by Reversible Succinylation. Mol. Cell.

[91] Sepa-Kishi D.M., Jani S., Da Eira D., Ceddia R.B. (2019). Cold acclimation enhances UCP1 content, lipolysis, and triacylglycerol resynthesis, but not mitochondrial uncoupling and fat oxidation, in rat white adipocytes. Am. J. Physiol. Cell Physiol..

[92] Motiejunaite J., Amar L., Vidal-Petiot E. (2021). Adrenergic receptors and cardiovascular effects of catecholamines. Ann. Endocrinol..

[93] Von Bank H., Hurtado-Thiele M., Oshimura N., Simcox J. (2021). Mitochondrial Lipid Signaling and Adaptive Thermogenesis. Metabolites.

[94] Fedorenko A., Lishko P.V., Kirichok Y. (2012). Mechanism of fatty-acid-dependent UCP1 uncoupling in brown fat mitochondria. Cell.

[95] Diaz-Gerevini G.T., Repossi G., Dain A., Tarres M.C., Das U.N., Eynard A.R. (2016). Beneficial action of resveratrol: How and why?. Nutrition.

[96] Wang Y., Zhao X., Lotz M., Terkeltaub R., Liu-Bryan R. (2015). Mitochondrial biogenesis is impaired in osteoarthritis chondrocytes but reversible via peroxisome proliferator-activated receptor γ coactivator 1α. Arthritis Rheumatol..

[97] Ray Hamidie R.D., Yamada T., Ishizawa R., Saito Y., Masuda K. (2015). Curcumin treatment enhances the effect of exercise on mitochondrial biogenesis in skeletal muscle by increasing cAMP levels. Metabolism.

[98] Tang X., Cui Y., Feng B. (2024). The chemical constituents and metabolite profiles of Huangqin decoction in normal and ulcerative colitis rats by UHPLC-Q-TOF/MS analysis. J. Pharm. Biomed. Anal..

[99] Ma M., Chen L., Tang Z., Song Z., Kong X. (2023). Hepatoprotective effect of total flavonoids from *Carthamus tinctorius* L. leaves against carbon tetrachloride-induced chronic liver injury in mice. Fitoterapia.

[100] Gu S., Xue Y., Liu X., Tang Y., Wang D., Wu D., Yao M., Xia Z., Yang S., Cai G. (2024). Clinical, pharmacology and *in vivo* studies of QingDai (indigo naturalis) promotes mucosal healing and symptom improvement in ulcerative colitis by regulating the AHR-Th17/Treg pathway. J. Inflamm..

[101] Huie L., Qiong W., Huijie A., Qiang Z., Wei D., Ying J., Siwei P., Huihui D., Aiwu L. (2025). Integrating bioinformatics with network pharmacology and experimental validation analysis to reveal the pharmacological mechanism of Ma-xing-shi-gan-tang in treating viral pneumonia. Phytomed. Plus.

